# Quantifying residue-specific conformational dynamics of a highly reactive 29-mer peptide

**DOI:** 10.1038/s41598-020-59047-7

**Published:** 2020-02-13

**Authors:** William R. Lindemann, Ethan D. Evans, Alexander J. Mijalis, Olivia M. Saouaf, Bradley L. Pentelute, Julia H. Ortony

**Affiliations:** 1 Department of Materials Science and Engineering, Massachusetts Institute of Technology, 77 Massachusetts Avenue, Cambridge, Massachusetts 02139 United States; 2 Department of Chemistry, Massachusetts Institute of Technology, 77 Massachusetts Avenue, Cambridge, Massachusetts 02139 United States; 3 Present Address: Department of Biological Engineering, Massachusetts Institute of Technology, Cambridge, Massachusetts 02139 United States; 4 Present Address: Department of Genetics, Harvard Medical School, Boston, Massachusetts 02115 USA; 5 Present Address: Department of Materials Science and Engineering, Stanford University, 496 Lomita Mall, Stanford, California 94305 United States

**Keywords:** Biological techniques, Biophysics, Chemistry

## Abstract

Understanding structural transitions within macromolecules remains an important challenge in biochemistry, with important implications for drug development and medicine. Insight into molecular behavior often requires residue-specific dynamics measurement at micromolar concentrations. We studied MP01-Gen4, a library peptide selected to rapidly undergo bioconjugation, by using electron paramagnetic resonance (EPR) to measure conformational dynamics. We mapped the dynamics of MP01-Gen4 with residue-specificity and identified the regions involved in a structural transformation related to the conjugation reaction. Upon reaction, the conformational dynamics of residues near the termini slow significantly more than central residues, indicating that the reaction induces a structural transition far from the reaction site. Arrhenius analysis demonstrates a nearly threefold decrease in the activation energy of conformational diffusion upon reaction (8.0 *k*_*B*_*T* to 3.4 *k*_*B*_*T*), which occurs across the entire peptide, independently of residue position. This novel approach to EPR spectral analysis provides insight into the positional extent of disorder and the nature of the energy landscape of a highly reactive, intrinsically disordered library peptide before and after conjugation.

## Introduction

**Figure 1:**
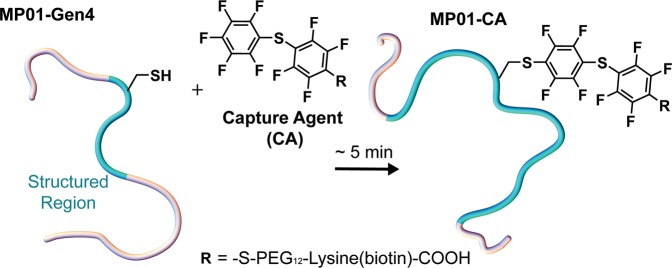
The MP01-Gen4 peptide reacts rapidly with a perfluoroarene capture agent (CA) via nucleophilic aromatic substitution (S_N_Ar) to form the complex MP01-CA in approx. 5 mins. The peptide backbone’s dynamic structure (illustrated here as a cartoon) is related to the high reactivity of MP01-Gen4 with perfluoroarenes.

Combinatorial, library-based strategies for discovering functional peptides have transformed biochemistry, enabling tremendous improvements in enzyme design, disease diagnosis, and drug development^1^. One prototypical example of sequences identified by combinatorial discovery is the family of MP peptides–molecules selected to undergo nucleophilic aromatic substitution (S_N_Ar) reactions via a single cysteine residue (Fig. 1)^2–4^. Their mild reaction conditions make reactive MP peptides optimal for bioconjugation^5–8^ while preserving orthogonality to other popular conjugation methods including click chemistry^9–11^, protein-facilitated approaches (such as the biotin-streptavidin interaction)^12–16^, and the use of peptide tags^17–20^. Bioconjugation tools have become essential technology, enabling controlled coupling of biomolecules for important diagnostic and therapeutic purposes. In the case of MPs, many features of their backbone dynamics and conformational behavior remain unknown because the residue-specific measurements required are difficult to achieve at low (μM) concentrations^2–4^.

Here we investigate MP01-Gen4, an abiotic 29-mer selected from among 5 × 10^13^ randomized peptides and subsequently optimized via experimental and computational methods^2,4^. The resulting sequence reacts rapidly with perfluoroarenes, demonstrating quantitative conversion in under five minutes (Fig. 1). Previously reported circular dichroism (CD) measurements show experimentally that MP01-Gen4 undergoes a random-coil-to-helix structural transformation upon interaction with a perfluoroarene probe^4,21^. Calculations from the PrDOS intrinsic disorder prediction tool suggested disorder in residues 1–7 and 24–29, and predictions using Rosetta software suggest the existence of transient α-helix-like order in residues near the center of the peptide, prior to S_N_Ar reaction^4^. Circular dichroism studies demonstrate that the peptide increases in helical content upon reaction, but neither these experiments nor PrDOS/Rosetta predictions could identify the residues involved. Thus, experimentally identifying the residues involved in this transition, and understanding the extent to which distinct regions of the sequence exhibit disorder or flexibility, is important for understanding the behavior of MP01-Gen4. Although this type of structural transition is common among natural sequences, its emergence from a library of abiotic peptides in the context of a non-biological reaction is noteworthy^22–24^. We aimed to identify the residues involved in this structural transition and to understand the relationship between the dynamic behavior of MP01-Gen4 and its structural transition^4^.

Conformational studies of peptides typically require residue-specific insight into dynamics. We acquired this information by introducing radical electron spin-labels at specific residues of MP01-Gen4 and performing electron paramagnetic resonance (EPR) spectroscopy to obtain rotational diffusion coefficients (inversely related to rotational correlation times) of the spin-label’s motion^25–28^. This motion primarily originates from conformational changes of the backbone, and its timescale depends on position, since more flexible regions of a peptide change conformation more rapidly^29^. We can therefore use this approach to map the flexibility of a sequence with residue-level resolution, even at micromolar concentrations^30–37^.

## Results and Discussion

### Rapid flow peptide synthesis enables incorporation of amino acid spin labels

We synthesized spin-labeled MP01-Gen4 using an Fmoc-protected amino acid whose R-group contains a nitroxide radical spin-label, TOAC (TOAC = 2,2,6,6-tetramethylpiperidine-*N*-oxyl-4-(9-fluorenylmethyloxycarbonyl-amino)-4-carboxylic acid)^38,39^. TOAC-containing peptides are desirable as EPR probes because they integrate intimately into the peptide backbone, providing an accurate measure of local dynamics. However, their synthesis remains challenging.

Overcoming TOAC’s steric limitations requires long coupling times and multiple couplings^40^. The speed and reliability of most amino acid couplings is improved by rapid-flow synthesis at elevated temperatures^41,42^. We adapted rapid-flow peptide synthesis to the preparation of TOAC peptides to enable reliable incorporation at arbitrarily chosen sites. TOAC positions were chosen to provide approximately regular spacing, by a systematic scan of alanine substitutions, which we used to identify locations where modifications would minimally perturb the reactivity of the peptide. In a two positions (5 and 7) we were willing to replace residues known to be important for reactivity, on the basis that we didn’t want to replace nearby charged residues.

### Conformational stabilization of the peptide’s termini

**Figure 2:**
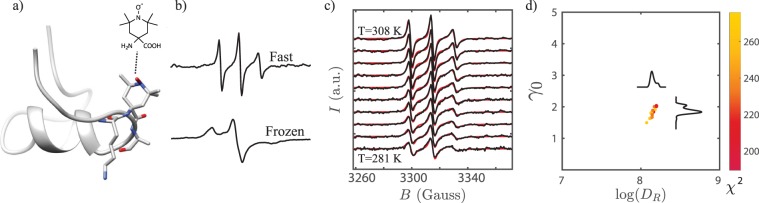
Experimental approach to dynamics measurements. (**a**) TOAC is embedded along the MP01-Gen4 backbone. (**b**) EPR line shapes of a TOAC-labeled MP01-Gen4 peptide at 308 K (top) and 150 K (bottom) indicate fast and slow rate of motion, respectively. (**c**) EPR spectra are fit for rotational diffusion rate at different temperatures. (**d**) The fitting function (${\chi }^{2}$) represents deviation between experimental data and a fitting model. Optimal values for fit parameters such as the log of the rotational diffusion coefficient (log(D_R_)) and the Gaussian line-broadening (*γ*_0_) are extracted from clusters of good fits near the global minimum of ${\chi }^{2}$.

We labeled each peptide (Fig. 2a) and measured its EPR spectrum. The line shapes of the spectra encode dynamics information (Fig. 2b). We fit our measurements at each probe position at ten temperatures, ranging from 280 K to 308 K (Fig. 2c), and measured distributions of good fits in order to quantify uncertainty (Fig. 2d). This strategy, described in the Methods section, enabled rotational diffusion rate, ${D}_{R}$, measurements at each site and temperature.

We introduced TOAC-substituted MP01-Gen4 to the perfluoroarene capture agent (CA) and observed rapid, near-quantitative conversion of MP01-Gen4 in almost every case (Table 1). These yields suggest that the incorporation of TOAC conserved the important features of MP01-Gen4 for enhanced reactivity. The only damaging substitution came at the lysine at position 20, which showed a reaction yield of 81% after replacement with TOAC. Interestingly, literature shows that substituting alanine into position 20 of a closely-related peptide enhances reaction rate^4^. Nonetheless, we kept this substitution to maintain approximately uniform TOAC spacings across the peptide.

**Table 1:** Conversion yields of the MP01-Gen4 reaction with perfluoroarene capture agent (CA) remain high for all ten spin labeled analogs of the peptide, with the exception of MP01-J20.

Designation	Sequence*	Conversion Yield†
MP01-Gen4	MNQKYKMAKA***C***FFAFLEHLKKRKLYPMSG	**
MP01-J3	MN**J**KYKMAKA***C***FFAFLEHLKKRKLYPMSG	92.8
MP01-J5	MNQK**J**KMAKA***C***FFAFLEHLKKRKLYPMSG	98.3
MP01-J7	MNQKYK**J**AKA***C***FFAFLEHLKKRKLYPMSG	97.3
MP01-J13	MNQKYKMAKA***C***F**J**AFLEHLKKRKLYPMSG	95.4
MP01-J16	MNQKYKMAKA***C***FFAF**J**EHLKKRKLYPMSG	93.0
MP01-J18	MNQKYKMAKA***C***FFAFLE**J**LKKRKLYPMSG	96.2
MP01-J20	MNQKYKMAKA***C***FFAFLEHL**J**KRKLYPMSG	80.8
MP01-J23	MNQKYKMAKA***C***FFAFLEHLKKR**J**LYPMSG	95.2
MP01-J27	MNQKYKMAKA***C***FFAFLEHLKKRKLYP**J**SG	99.6
MP01-J29	MNQKYKMAKA***C***FFAFLEHLKKRKLYPMS**J**	94.7

*J is the amino acid spin-label TOAC; ***C*** is the reactive cysteine.

^†^Calculated from integrated LCMS peak intensity (Fig. S1–2).

**Quantitative conversion; details reported elsewhere^4^.

**Figure 3:**
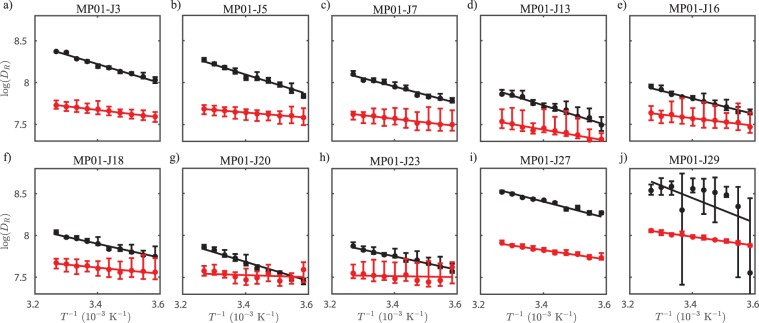
Arrhenius plots of residue-specific dynamics of MP01-Gen4 before (black) and after (red) reaction with MP01-Gen4. Y-Axes reflect conformational dynamics, indicated by rotational diffusion rates, determined by EPR analysis of residue-specific spin labels. Unreacted peptides diffuse more rapidly than the reacted peptides, especially near the termini. Due to the high error associated with MP01-J29, arising from low spectral intensity, a fit is not provided for these data.

We measured EPR spectra of TOAC peptides after conjugation with the CA. In Fig. 3, we report the rotational diffusion coefficients ($\log ({D}_{R})$) of each spin-label site of MP01-Gen4, both before (black) and after (red) conjugation. MP01-Gen4 experiences a sharp change in dynamics upon reaction with its target, behaving more like a rigid, structured molecule.

**Figure 4:**
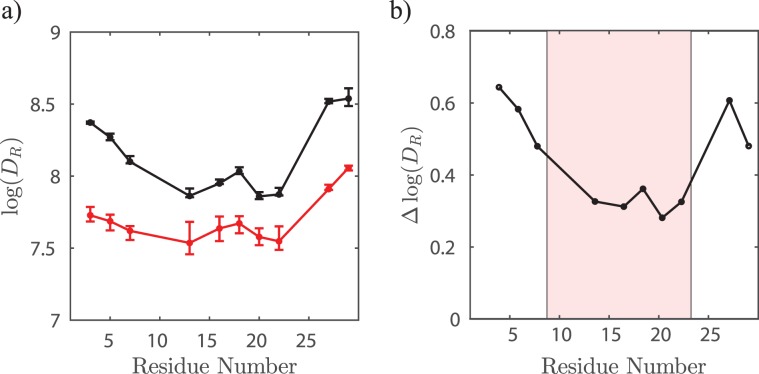
The initially disordered region experiences a greater change in dynamics upon reaction. (**a**) Rotational diffusion at each probe position, collected at 35 °C for unlabeled (black) and labeled (red) MP01. (**b**) Change in rotational diffusion upon reaction. Resides predicted to be ordered before the reaction (shaded region), experience a smaller and consistent $\triangle \log ({D}_{R})$ of 0.32 ± 0.03, compared to other residues, located in initially disordered regions.

MP01-Gen4’s rate of conformational change varies strongly with position (Fig. 3). For instance, dynamics of the unreacted peptide at residue 27 (Fig. 3i) are greater than at residue 23 (Fig. 3h), as shown by the overall higher rotational diffusion rates across the temperature sweep. Residues near the termini of MP01-Gen4 change conformation more rapidly than the central residues (Fig. 4). The five TOAC positions located within the central region show similar rates of dynamic motion at any given temperature ($\log ({D}_{R})\approx 8$ at 310 K). Dynamics at the other five positions are faster – especially residues 27 and 29. Upon reaction with CA, the rate of dynamic motion slows dramatically throughout MP01-Gen4 (Fig. 4). This change is most pronounced in non-central residues, and is almost constant in the central region. The most drastic decline in dynamics upon binding occurs in the terminal residues (3, 5, 7, 27 and 29), suggesting that these undergo the greatest structural change.

### Connecting the structural transition with the activation energy of diffusion

**Figure 5:**
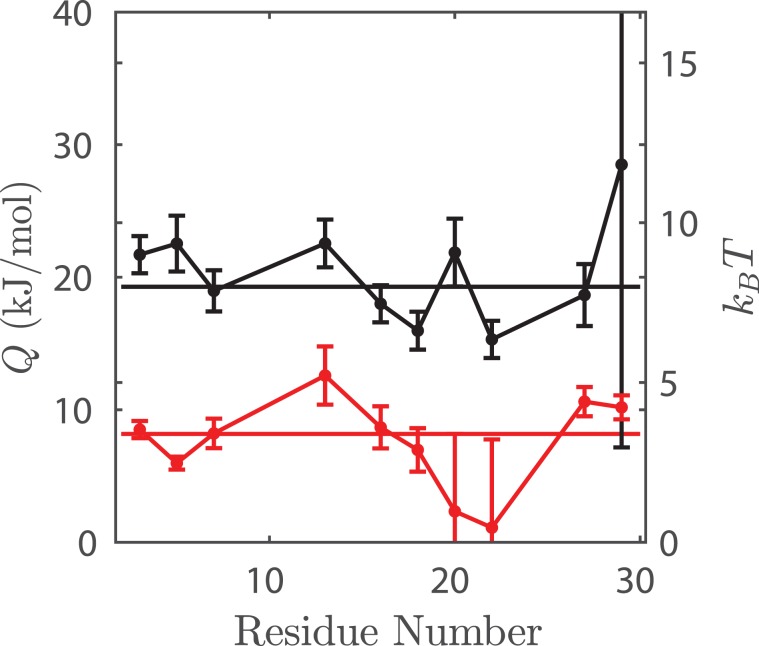
Reaction is accompanied by a significant drop in diffusional activation energy. Activation energy (*Q*) of rotational diffusion vs. TOAC position in unreacted (black) and reacted (red) MP01. Average *Q* (8.0 *k*_*B*_*T* unreacted, 3.4 *k*_*B*_*T* reacted) is plotted as a line. Notably, *Q* is relatively independent of residue number but drops by >60% upon conjugation. Due to the high error associated with MP01-J29, arising from low spectral intensity, *Q* is not provided for this peptide.

The activation energy (${\rm{Q}}$) of rotational diffusion represents the energetic barrier to conformational change of the peptide backbone. Using the Arrhenius plots (Fig. 3), we extracted activation energies of rotational diffusion of each peptide, which we plotted as a function of residue number (Fig. 5). Upon reaction, MP01-Gen4 exhibits a global >60% drop in ${\rm{Q}}$ (from an average of 8.0 ${k}_{B}T$ to 3.4 ${k}_{B}T$ at $T=298$ K). The observed positional independence of ${\rm{Q}}$ implies that dynamic motion occurs because of global changes in conformation, rather than local effects.

At a given temperature and in the absence of significant structural changes, we would expect ${D}_{R}$ and ${\rm{Q}}$ to scale inversely, according to the Arrhenius equation ${D}_{R}={D}_{0}\exp (\,-\,\frac{Q}{RT})$. However, despite the positional-independence of ${\rm{Q}}$, we observed substantial variation in ${D}_{R}$ as a function of position within a peptide (Fig. 4). This occurs because the Arrhenius prefactor, ${D}_{0}$, depends upon the degree to which each conformational change displaces the nitroxide. This explains why, within a given peptide, ${D}_{R}$ is greater in regions which are more flexible – despite the similarity of their ${\rm{Q}}$ –values. Changes in ${D}_{0}$ also explains why, upon reaction with CA, MP01-Gen4 experiences a decrease in ${D}_{R}$ despite also experiencing a decrease in $Q$. In this case, the molecule becomes not only more helical, but also larger, reducing the amplitude of vibrations that displace the nitroxide radical.

In spin-labeled EPR experiments, rotational diffusion is known to occur because the peptide diffuses through distinct conformations. Therefore, temperature dependence of conformational motion is related to the peptide’s conformational free energy landscape^43–45^. For peptides and proteins, this energy landscape is rough, populated by small kinetic traps^46–49^. As the peptide diffuses through its conformations, it must hop between these traps. Therefore, by measuring $\log ({D}_{R})$ we sample the subset of those conformational changes of the backbone which move the nitroxide probe. We hypothesize that in our peptides, the activation energy of rotational diffusion must scale with the average energy barrier between distinct peptide conformations.

Typically, short proteins demonstrate energy landscape roughness values in the range of 0–5 ${k}_{B}T$. Our observed activation energies of $8.0\,{k}_{B}T$ before conjugation and $3.4\,{k}_{B}T$ after conjugation fall above this range – though still well within the diffusional regime. Other EPR experiments demonstrate similar activation behavior in proteins, noting that these values suggest H-bond formation between the nitroxide probe and the hydration shell^44^. Therefore, our observed ${\rm{Q}}$ values suggest that we are sampling the subset of conformational changes with sufficient energy to break this H-bond.

The decrease in ${\rm{Q}}$ upon MP01-Gen4 conjugation is significant. In the absence of other factors, the activation energy for diffusive motion of short polymers typically increases with molecular weight, due to increased internal friction^50,51^. Higher activation energies correspond to rougher energy landscapes, which occur when no single configuration adequately satisfies all of the intramolecular interactions necessary to fully stabilize it^52^. Rougher energy landscapes tend to correspond to greater intrinsic disorder, since disordered peptides experience greater structural change between quasi-stable states. Therefore, this decrease in ${\rm{Q}}\,$demonstrates that reaction with a perfluoroarene stabilizes multiple chemical sites on the MP01-Gen4 peptide that cannot otherwise be simultaneously satisfied^52^. These stabilizing interactions could form directly with the perfluoroarene, or because structural reorientation enables stronger intra-chain interactions elsewhere within the peptide. Energy landscape roughness cannot be used as a proxy for depth of the energy basin, and does not describe a peptide’s overall stability or absolute state of disorder. However, in the case of MP01-Gen4, the decrease in roughness likely occurs because the system finds a more stable secondary structure. This methodology provides a novel approach for describing conformational changes within a peptide.

## Conclusions

We performed the first flow-synthesis of TOAC peptides in order to study the residue-specific dynamic behavior of MP01-Gen4, a peptide designed to react with perfluoroarenes for bioconjugation chemistry. Through EPR analysis, we found that while native MP01-Gen4 is flexible and largely disordered, upon reaction with the perfluoroarene the peptide becomes significantly more rigid. Further, we identified the residues involved in the structural change, and designate the expansion of the central helical region towards the termini as its origin. Based on new physical insights, we demonstrated that a >60% decrease in the activation energy of diffusion upon reaction of MP01-Gen4 with a perfluoroarene capture agent suggests a decrease in the conformational energy landscape roughness. Thus, we conclude that MP01-Gen4 experiences a structural change upon reaction, especially in the initially-unstructured region near the N-terminus, suggesting a disorder-to-order transition upon reaction. Our results identify frustration and disorder of unreacted chains as a potentially important parameter in designing reactive peptides, and demonstrates the broad potential of EPR spectral simulations and Arrhenius analysis for studying the relationship between peptide structural transitions and reactivity. These insights could be used to design more effective screening libraries for bioconjugation.

## Methods

### TOAC peptide synthesis

Peptides and the perfluoroarene capture agent (CA) were synthesized according to literature, using ChemMatrix H-rink amide resin (0.49 meq/g) on the 0.1 mmol scale^2,42^. Flow-synthesis of standard amino acids uses a DMF solution of 0.2 M amino acid, 0.17 M activating agent and 5% (v/v) DIPEA flushed over the sample at 80 mL/min for 15 s, followed by DMF washing and deprotection using DMF 20% piperidine. TOAC was coupled using 50 mM Fmoc-TOAC, 47.5 mM HATU, and 10% DIPEA at a rate of 40 mL/min for 15 s, followed by the usual washing and Fmoc-deprotection steps. Due to steric limitations of the TOAC, the kinetics of coupling natural amino acids to resin-bound TOAC proved to be exceptionally slow. To bypass this problem, we couple the sterically-hindered post-TOAC residue using 0.2 M amino acid, 0.14 M activating agent and 10% DIPEA pumped at 10 mL/min for 10 min, followed by the usual wash and deprotection steps. All subsequent residues were coupled normally. Completed peptides were cleaved for 2 h at RT using (90% TFA, 5% water, 5% TIPS v/v), a cleavage cocktail for TOAC peptides^40^. The resulting peptides were then precipitated and washed 3x in diethyl ether (−80 °C), before drying under vacuum. The dried product was dissolved and purified by reverse phase high performance liquid chromatography (HPLC). Synthetic yields for each peptide, calculated from the crude mass collected, are reported in the supplement (Table S1).

### LCMS analysis

The purity of all peptides was analyzed by liquid chromatography mass spectrometry (LCMS) using an Agilent 6520 ESI-Q-TOF mass spectrometer. For convenience, solutions A and B are defined as follows: A – water, 0.1% formic acid; D – acetonitrile, 0.1% formic acid. LCMS was carried out according to the following steps: in the range of 0–2 min, a 95% A - 5% B wash; in the range of 2–11 min, a 5–65% B linear ramp; and in the range of 11–12 min, a 65% B. We used a flow rate of 0.8 mL/min on a Zorbax 300SB C3 column (2.1 × 150 mm, 5 μm), at 40 °C. MS was performed by positive electrospray ionization (ESI). Observed masses were reported by averaging the major peak in the total ion current (TIC).

### Preparative HPLC

Crude peptides were purified by reverse phase high performance liquid chromatography (HPLC). Solutions C and D are defined as follows: C – water, 0.1% trifluoroacetic acid; D – acetonitrile, 0.1% trifluoroacetic acid. Peptides were dissolved in 50% C, 50% D and loaded onto an Agilent Zorbax C3 column (21.2 × 250 mm, 7 μm). HPLC was carried out at a flow rate of 5 mL/min according to the following steps: in the range of 0–5 minutes, a 95% C – 5% D wash; in the range of 5–80 min, a 5–45% C linear ramp; and in the range of 80–85 min, a 45% C wash.

### EPR sample preparation

All EPR samples were prepared by injecting 10 μL solutions of peptide in 1x phosphate buffer solution (PBS) at a concentration of 45 μM into a PTFE capillary tubes, sealed with Crytoseal resin. S_N_Ar reactions were performed at 45 μM peptide concentration in PBS at RT for 15 min, with CA in 5x molar excess. Potassium hexacyanoferrate(III) (K_3_Fe(CN)_6_) was added to all samples before EPR analysis to reverse the reduction of nitroxides by TFA. The maximum K_3_Fe(CN)_6_ concentration that did not result in detectable peptide degradation was used in each case, and no subsequent purification efforts were made since neither unreacted hydroxylamines nor K_3_Fe(CN)_6_ interfere with the nitroxide EPR signal. In the case of unreacted peptides, 0.2 equiv. K_3_Fe(CN)_6_ was used for all analysis. In the case of the reacted peptides, 1 equiv. K_3_Fe(CN)_6_ was used for all analysis. The exception in both cases was the sequence MP01-J29. This peptide is less stable in the presence of K_3_Fe(CN)_6_, so none was added to unreacted MP01-J29 and 0.2 equiv. were used for EPR analysis of the reacted peptide. The reduction of the nitroxide in MP01-J29 increased the uncertainty of the fit of unreacted MP01-J29. After EPR, each sample was recovered and analyzed by LCMS.

### EPR experiments

Continuous wave electron paramagnetic resonance (cw-EPR) spectra were collected at X-band (9.43 GHz) using a Bruker EMX + with a variable temperature unit. Spectra were collected over 150 G field sweep with center field at B = 3315 G, with attenuation of 15 dB and modulation amplitude of 1.5. EPR spectra of a background sample containing only PBS and K_3_Fe(CN)_6_ were subtracted from each peptide spectrum. Variable temperature spectra of each sample were collected in the range of 275–310 K, in increments of 5 K. We verified by LCMS that each peptide was undamaged by the heating process and that they reacted completely with the perfluoroarene target, demonstrating that their functionality was retained.

### EPR fitting

Initial fitting of each sample at 150 °C was carried out to determine hyperfine ($A$) and electron ($g$) tensors using the pepper function in Easyspin^28^. Since frozen spectra were identical under scaling, regardless of the position of TOAC, the fitted tensor components of ${g}_{xx}=2.0081$, ${g}_{yy}=2.0051$, ${g}_{zz}=2.0020$, ${A}_{\perp }=5.13$ G, and ${A}_{\parallel }=37.6$ G were assigned to all samples during EPR fitting at higher temperatures.

Analyses of higher-temperature EPR data were carried out using non-linear least squares analysis via the NLSL program to perform Levenberg-Marquardt curve-fitting^27,29^. We fit the data for the base 10 logarithm of rotational diffusion rate, $\log ({D}_{R})$, the Gaussian line-broadening, ${\gamma }_{0}$, and the ${c}_{20}$ ordering parameter, using the microscopic order, macroscopic disorder (MOMD) model with 50 orientations. Monte-Carlo variation of initial fit parameters was carried out with an in-house MatLab script within a reasonable physical range, adding a random Gaussian noise to each data point during each iteration. The mean of this Gaussian noise distribution was zero, and the standard deviation was taken from a region of the spectrum containing no TOAC intensity (where all signal arose from instrument noise). We performed 500 fits for each of the 200 spectra, choosing parameters in the range $6.5 < \log ({D}_{R}) < 9$, ${10}^{-3} < {\gamma }_{0} < 8$ and $-5 < {c}_{20} < 8$. Fits converging outside this range were discarded. $\log ({D}_{R})$ values were computed as the average value of good fits, inversely weighted by the fitting error ${{\rm{\chi }}}^{2}$, and 95% confidence intervals were computed using the distribution of fits with ${{\rm{\chi }}}^{2} > 1.5\ast {{\rm{\chi }}}_{{\rm{\min }}}^{2}$, where ${{\rm{\chi }}}_{{\rm{\min }}}^{2}$ is the ${{\rm{\chi }}}^{2}$ of the global best fit. This approach incorporates the error associated with overfitting into the confidence interval calculation. Overfitting is more common in slower-moving spectra, where a broader range of parameters can give rise to similar spectra. Activation energy ($Q$) was calculated by linear fitting of Arrhenius plots, using the equation:

$$\log ({D}_{R})=\,\log ({D}_{0})-\frac{Q\,\log (e)}{RT}$$

where ${D}_{0}$ is a constant, $R$ is the universal gas constant, $e$ is Euler’s number, and $T$ is temperature.

## Supplementary information

Supplementary Information.
