# Supplementary material for: Quantifying residue-specific conformational dynamics of a highly reactive 29-mer peptide
Source: Sci Rep. 2020 Feb 13;10:2597. doi: 10.1038/s41598-020-59047-7 (PMC7018720; doi:10.1038/s41598-020-59047-7)
Supplement: Supplementary file 1 — Supplementary Information. [file 41598_2020_59047_MOESM1_ESM.docx]

Table of Contents

[1. Supplemental Methods 2](#_Toc23426953)

[1.1 Materials 2](#_Toc23426954)

[1.2 Fitting method 2](#_Toc23426955)

[2. LCMS results 4](#_Toc23426956)

[2.1 LCMS Impurities and interpretation 4](#_Toc23426957)

[2.2 Purity data 5](#_Toc23426958)

[2.3 Conversion Yields from LCMS Results 6](#_Toc23426959)

[3. EPR Analysis 9](#_Toc23426960)

[3.1 Potential reasons for variation in activation behavior 9](#_Toc23426961)

[3.2 EPR spectral fits 11](#_Toc23426962)

[4. Mass Spectra of Unreacted Peptides 21](#_Toc23426963)

[5. Mass Spectra of Reacted Peptides 31](#_Toc23426964)

[6. References 41](#_Toc23426965)

# 1. Supplemental Methods

## 1.1 Materials

Peptides and the perfluoroarene capture agent (CA) were synthesized according to literature.^1^ 1-[Bis(dimethylamino)methylene]-1H-1,2,3-triazolo[4,5-b]pyridinium 3-oxid hexafluorophosphate (HATU), Fmoc-L-Ala-OH, Fmoc-L-Cys(trt)-OH, Fmoc-L-Glu(tBu)-OH, Fmoc-L-Phe-OH, Fmoc-Gly-OH, Fmoc-L-His(Boc)-OH Fmoc-L-Lys(Boc)-OH, Fmoc-L-Leu-OH, Fmoc-L-Met-OH, Fmoc-L-Asn(Trt)-OH, Fmoc-L-Pro-OH, Fmoc-L-Gln(Trt)-OH, Fmoc-L-Arg(Pbf)-OH, Fmoc-L-Ser(tBu)-OH, Fmoc-L-Tyr(tBu)-OH and Fmoc-TOAC-OH were purchased from Chem-Impex International. H-rink-amide ChemMatrix Hyr resin was obtained from PCAS BioMatrix, Inc. (7-Azabenzotriazol-1-yloxy)tripyrrolidinophosphonium hexafluorophosphate (PyAOP) was purchased from P3 BioSystems. *N,N*-dimethylformamide (DMF), acetonitrile (ACN) and diethyl ether were purchased from VWR (Radnor, PA). N,N-diisopropylethylamine (DIPEA), formic acid (FA), 10x phosphate buffered saline (PBS), trifluoroacetic acid (TFA) and triisopropylsilane (TIPS) were obtained from Sigma-Aldrich. Potassium hexaferrocyanate (III) (K_3_Fe(CN)­_6_) was purchased from Alfa-Aesar.

Alltech low pressure polytetrafluoroethane (PTFE) tubing and Leica BioSystems Crytoseal capillary tube sealant were purchased from Fisher-Scientific. Liquid nitrogen was purchased from Airgas. Wilmad 4x250 mm quartz glass EPR tubes were purchased from Cambridge Isotope Laboratories. Water (18.2 MΩ) was purified using a Milli-Q Direct 8 system.

## 1.2 Fitting method

We have observed that when fitting EPR spectra using this model, the $\chi^{2}$ function is rugged, meaning that the global minimum of $\chi^{2}$ is located within a highly turbulent region of the function, and may be found somewhere within a cluster of good fits with similar $\chi^{2}$ values. This behavior has been reported in other systems, and is exacerbated by the presence of experimental noise, which causes further uncertainty in the parameters of the best fit.^2^ The greatest source of error is a tendency towards overfitting near the global minimum of $\chi^{2}$. Even when fitting simulated data sets, this causes broadening of the $\chi^{2}$ minimum, which means that this method carries an inherent resolution limit.

Rather than reporting the absolutely optimal value of $log\left( D_{R} \right)$ (the one giving the global minimum of $\chi^{2}$), we report the median value of all ‘sufficiently good fits’ ($\chi^{2}<2\chi_{min}^{2}$) identified during our Monte Carlo process, weighted by fit quality (${1/\chi}^{2}$). This improves the self-consistency of fits, and allows us to produce histograms of good fits for each spectrum, as well as error-bars containing 95% of good fits. For each spectrum, we checked the histogram to make sure that multiple, equally good clusters of fits were not identified, since this would skew the error bars and median values. The value $\chi^{2}<2\chi_{min}^{2}$ was chosen to exclude data clustered around inferior fits. Since errors in measured $log\left( D_{R} \right)$ values are almost certainly correlated, random resampling would overstate error in the activation energy ($E_{a}$). Therefore, to estimate uncertainty in $E_{a}$, we used the standard error calculated from the linear fit.

We performed this analysis using a suite of in-house MatLab programs designed to work with NLSL (available publically from ACERT, <https://www.acert.cornell.edu/>).^3^ In most cases, the fits computed by this method were excellent (Fig. S4-13). However, the NLSL algorithm can become unreliable at slow rates of rotational diffusion, and in several such cases (specifically, reacted MP01-J3, J5, and J23) the fits deviate slightly from experimental data. However, by randomly sampling the parameter space and resampling data, we ensured that we obtained the best possible agreement between our model and the experiment. Moreover, the results were self-consistent, the activation energies agreed with those computed from the other seven reacted peptides, and we hesitate to incorporate extraneous fit parameters into our analysis because of concerns about over-fitting the data. Finally, we note that the reaction yield in MP01-J23 was lower than most of the other reactions (83%) and that this may have affected fit. We therefore chose to accept these fits as reasonable, opting for consistency in our general fitting method over the refinements we might be able to make by the incorporation of additional fit parameters or MOMD orientations.

# 2. LCMS results

## 2.1 LCMS Impurities and interpretation

In the case of MP01-J7, MP01-J13 and MP01-J27, a small quantity of the C-terminal glycine deletion product persisted during purification by HPLC. This is noted in the LCMS profiles of the labeled product after reaction with K_3_Fe(CN)­_6_ and EPR analysis, since the nitroxide variant elutes earlier than the hydroxylaminated variant, giving rise to a small, secondary peak. In LCMS of the unreacted peptides, this was not noted because of overlap with the primary peak. In each of these cases, the target product is by far the most dominant in the LCMS profiles, and in any case, the loss of the C-terminal glycine likely only produces a marginal difference in the dynamic behavior of other regions of the sequence. A C-terminal TOAC-deletion product in MP01-J29 appears for the same reason, but is less noteworthy because it cannot produce a nitroxide signal.

## 2.2 Crude yields and purity data

**Table S1.** Crude yields (mg) are reported for each peptide (column 2), along with masses expected/*observed* for the peaks present in the LCMS traces in Figures S1 (columns 3-4) and S2 (columns 5-7). In most cases, hydroxylamine signal is dominant, due to reduction of the nitroxide in acidic conditions before/during the LCMS scan. A minor peak, corresponding to the nitroxyl version of the C-terminal glycine deletion product, appeared in a few spectra – however, this is always a minority product and probably had a negligible impact on EPR analysis. Observed masses are calculated using the [M+3H]^3+^ charge state for the unlabeled peptides, and the [M+4H]^4+^ charge state for the labeled peptides

| Name | Crude Yield (mg) | Hydroxylamine | Nitroxide | Labeled Hydroxylamine | Labeled Nitroxide | Labeled Glycine Deletion |
| --- | --- | --- | --- | --- | --- | --- |
| MP01-J3 | 131 (69%) | 3576.89/*3576.87*^*^ | -- | 4891.35/*4891.35*^*^ | -- | -- |
| MP01-J5 | 50 (26%) | 3541.88/*3541.88*^*^ | 3540.88/*3540.88* | 4856.35/*4856.35*^*^ | 4855.34/*4855.34*^**^ | -- |
| MP01-J7 | 64 (33%) | 3573.91/*3573.92*^*^ | 3572.90/*3572.90* | 4888.37/*4888.34*^*^ | 4887.36/*4887.39*^**^ | 4830.34/*4830.41* |
| MP01-J13 | 110 (59%) | 3557.88/*3557.91*^*^ | 3556.87/*3556.87* | 4872.34/*4872.36*^*^ | -- | 4814.31/*4814.29* |
| MP01-J16 | 82 (41%) | 3591.86/*3591.86*^*^ | 3590.85/*3590.85* | -- | 4905.32/*4905.35*^*,†^ | -- |
| MP01-J18 | 56 (30%) | 3567.89/*3567.88*^*^ | -- | 4882.35/*4882.31*^*^ | 4881.34/*4881.30* | -- |
| MP01-J20 | 81 (42%) | 3576.85/*3576.85*^*^ | 3575.84/*3575.84* | 4891.32/*4891.18*^*^ | 4890.31/*4890.17* | -- |
| MP01-J23 | 62 (32%) | 3576.85/*3576.83*^*^ | -- | 4891.32/*4891.22*^*^ | 4890.31/*4890.21* | -- |
| MP01-J27 | 100 (53%) | 3573.91/*3576.92*^*^ | -- | 4888.37/*4888.31*^*^ | -- | 4830.34/*4830.22* |
| MP01-J29 | 20 (10%) | 3647.92/*3647.94*^*^ | -- | 4962.39/*4962.36*^*^ | -- | -- |
| ^*^ This is the principle peak observed in the LCMS traces shown in figure S1-S2  ^**^ The nitroxide LCMS trace overlaps the hydroxylamine trace, but appears to be the minor product  ^†^ In all but this case, the primary product contains the hydroxylaminated version of the TOAC residue, due to reducing conditions prior to/during loading onto the LCMS column. In this case, the true nitroxide form (which differs by the mass of an H_1_ atom) dominated – either because the sample was loaded relatively quickly or because proximity to the labeled cysteine more effectively protected this nitroxyl radical from reduction. | | | | | | |

## 2.3 Conversion Yields from LCMS Results


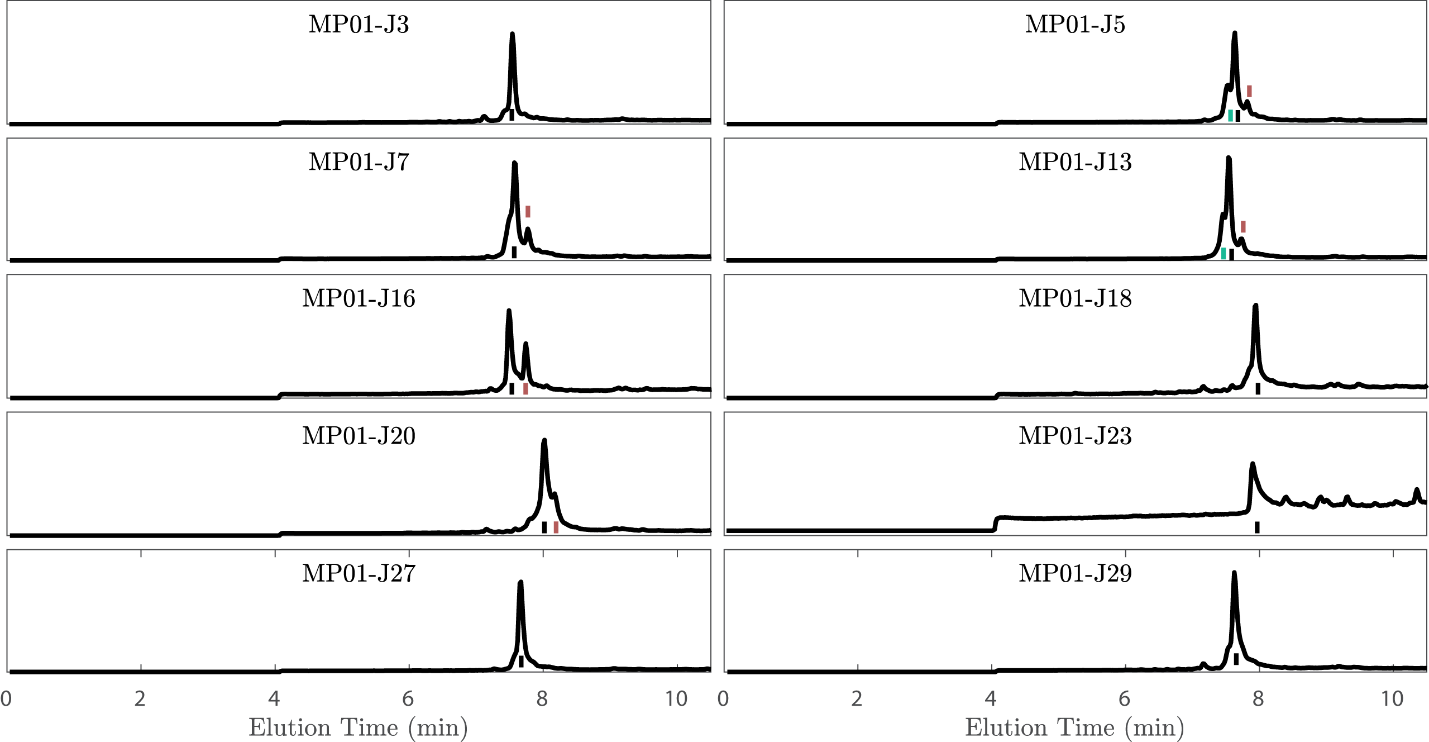


**Figure S1.** LCMS elution profiles of the peptides before S_N_Ar reaction and after EPR analysis. Since the samples were diluted in a solution containing TFA, and because the LCMS column contains FA, the product often appears in both its nitroxyl and its hydroxylamine forms, and these are marked by red and black lines, respectively. The presence of a small dimer peak was usually noted. This is marked by a green line when separate from the principle peak. Reaction yields were estimated by comparing the integrated peak intensities of the unreacted species shown here (via integration/addition of both hydroxylamine and nitroxyl peaks) and after undergoing reaction (Fig. S2).


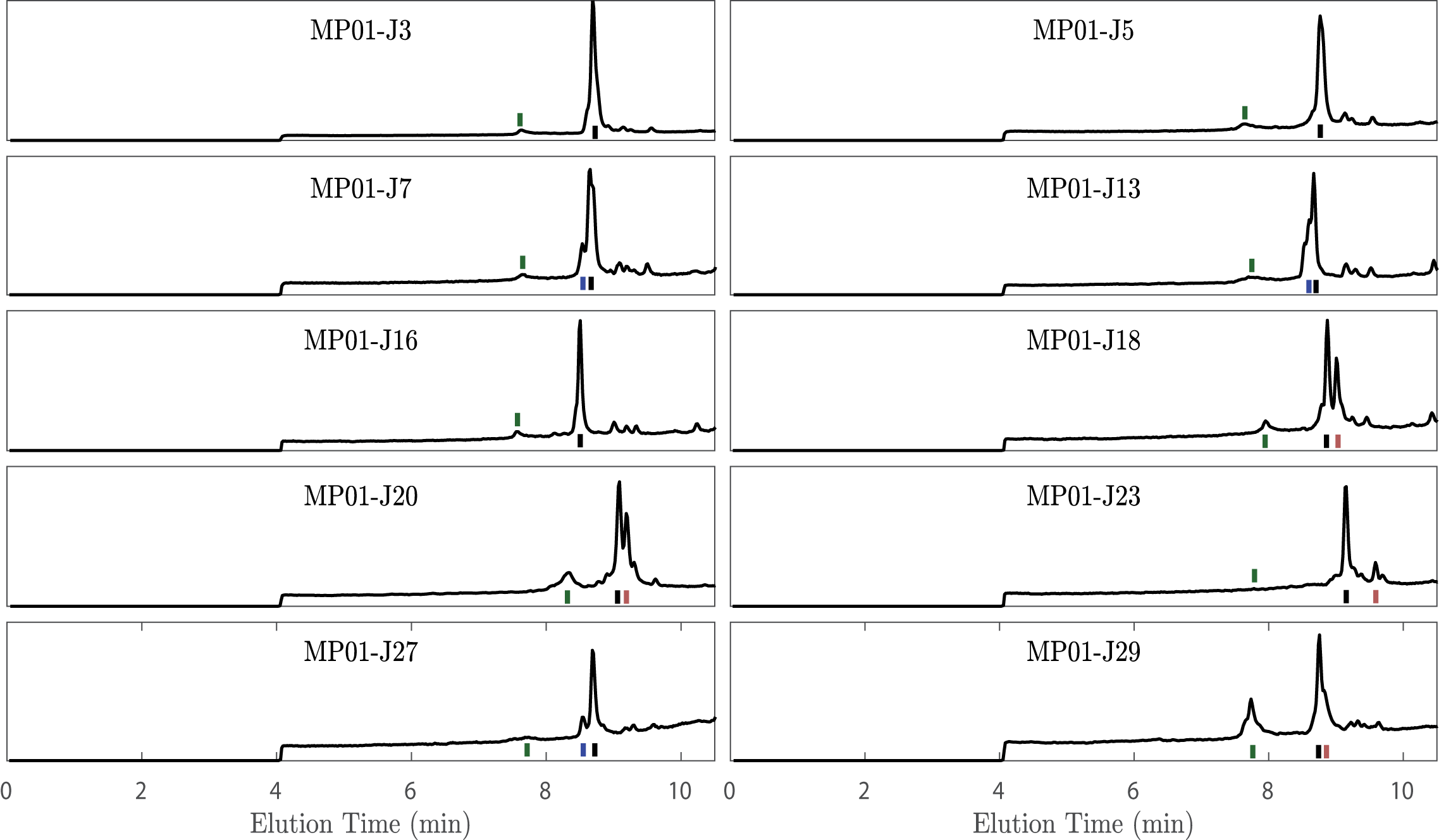


**Figure S2.** LCMS elution profiles of the peptides following S_N_Ar reaction and EPR analysis. Since the samples were diluted in a solution containing TFA, and because the LCMS column contains FA, the product often appears in both its nitroxyl and its hydroxylamine forms, and these are marked by red and black lines, respectively. The unreacted peptide peak is marked with a green line. Reaction yields were estimated by comparing the integrated peak intensities of the unreacted species shown here (via integration/addition of both hydroxylamine and nitroxyl peaks) and the same peak prior to reaction (Fig. S1). In MP01-J7, J13 and J27, a minor glycine deletion product was noted (blue) that is invisible in the elution profiles prior to S_N_Ar reaction, due to overlap with the principle product.


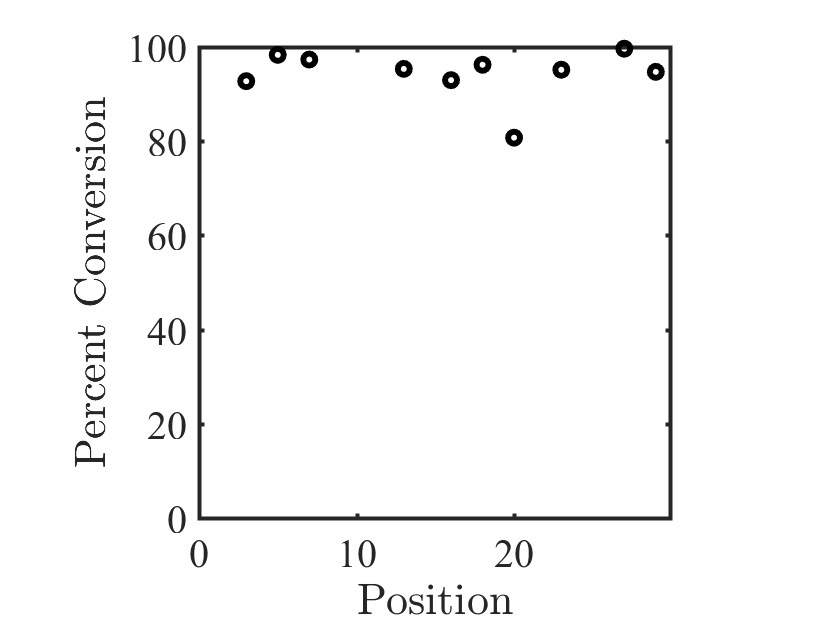


**Figure S3.** Estimated conversion yield of the S_N_Ar reaction, computed by the peak-integration of the LCMS traces shown in Figure S1. Precise values are reported in Table S1.

# 3. EPR Analysis

## 3.1 Potential reasons for variation in activation behavior

Residue-specific variation in $Q$ originates from three sources: site-specific variations in dynamic behavior, the uncertainty associated with spectral fitting, and the replacement of other amino acids with TOAC. We addressed fit uncertainty through Monte Carlo analysis of fit parameters, providing meaningful uncertainty estimates and avoid overfitting. Despite the care with which we selected positions for TOAC substitution, we did observe minor, sequence-specific differences in peptide reactivity. These substitutional differences likely account for most of our variation in activation energy. However, the self-consistency of both reactivity and spectral measurements indicates that the TOAC did not seriously perturb the behavior of the native MP01-Gen4 sequence (Table 1).

Finally, variations arise because although conformation is a global variable, only conformational changes causing TOAC reorientation will affect an EPR spectrum. In other words, the apparent activation energy only corresponds to the subset of conformational changes that cause spin-label motion, and these changes may require traversal of an average energy barrier different than the energy barrier for overall conformational change. This means that, unlike every other technique to measure energy landscape roughness, EPR may sample a specific subspace of the conformational energy landscape, rather than sampling the entire landscape. According to this hypothesis, a protein containing both an unstructured, flexible region and a highly structured, immobile region could experience differences in activation energy of rotational diffusion, depending on whether TOAC appears in the rigid part or the flexible part. While this effect is likely minor in small peptides like MP01-Gen4, it nonetheless explains some of the variability observed between peptides with differently positioned labels.

## 3.2 EPR spectral fits


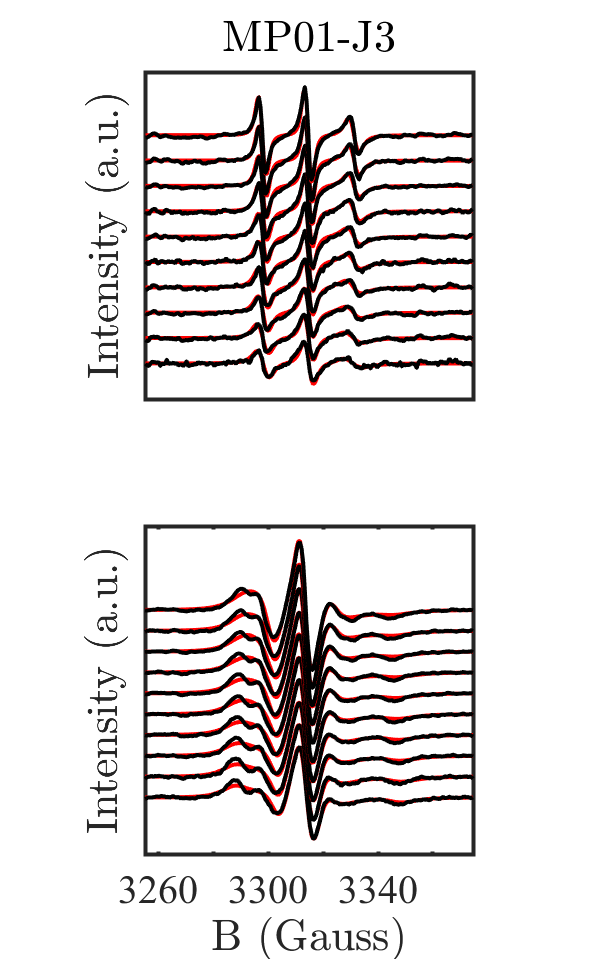


**Figure S4.** EPR spectra and best fits for unreacted (top) and reacted (bottom) MP01-J3.


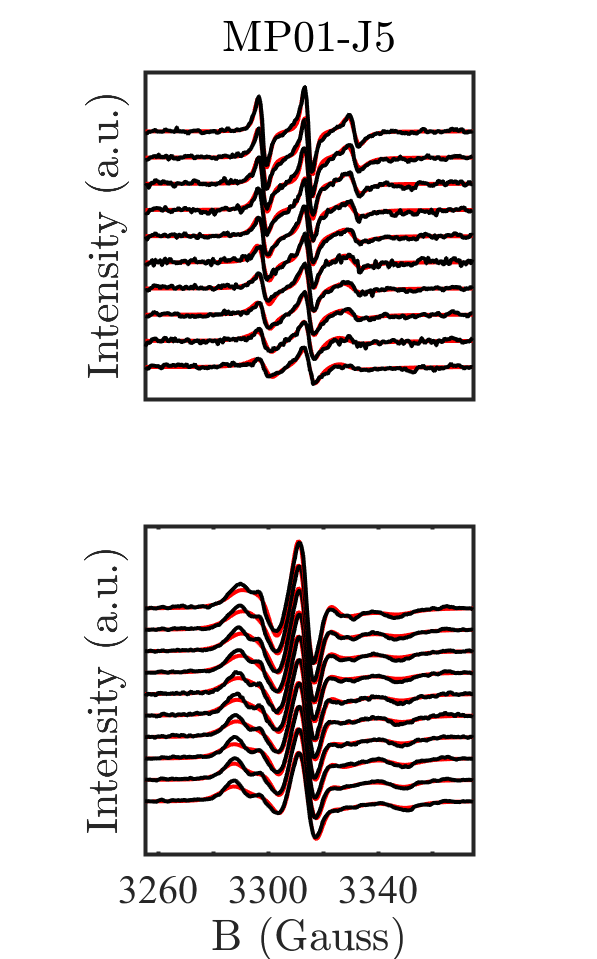


**Figure S5.** EPR spectra and best fits for unreacted (top) and reacted (bottom) MP01-J5.


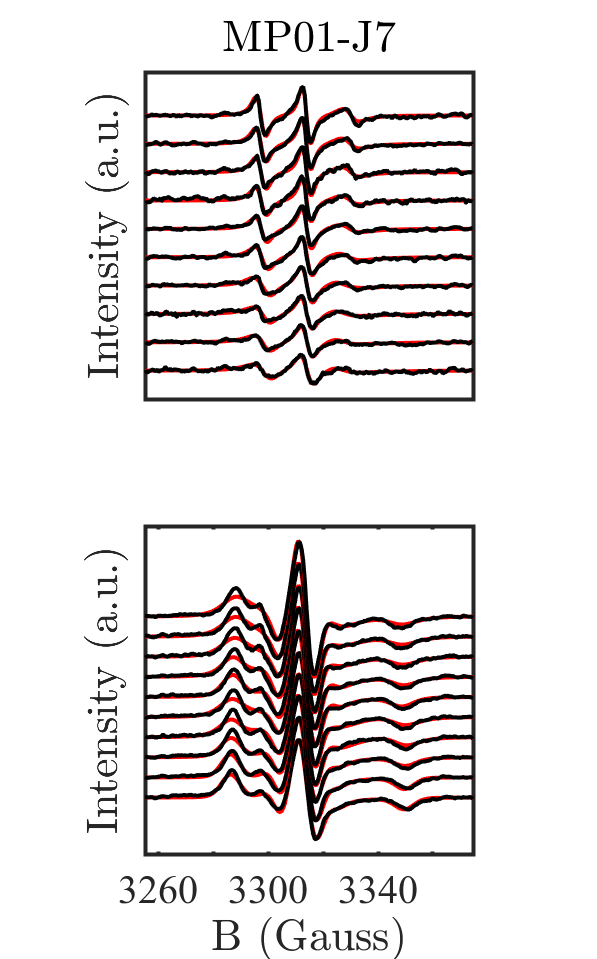


**Figure S6.** EPR spectra and best fits for unreacted (top) and reacted (bottom) MP01-J7.


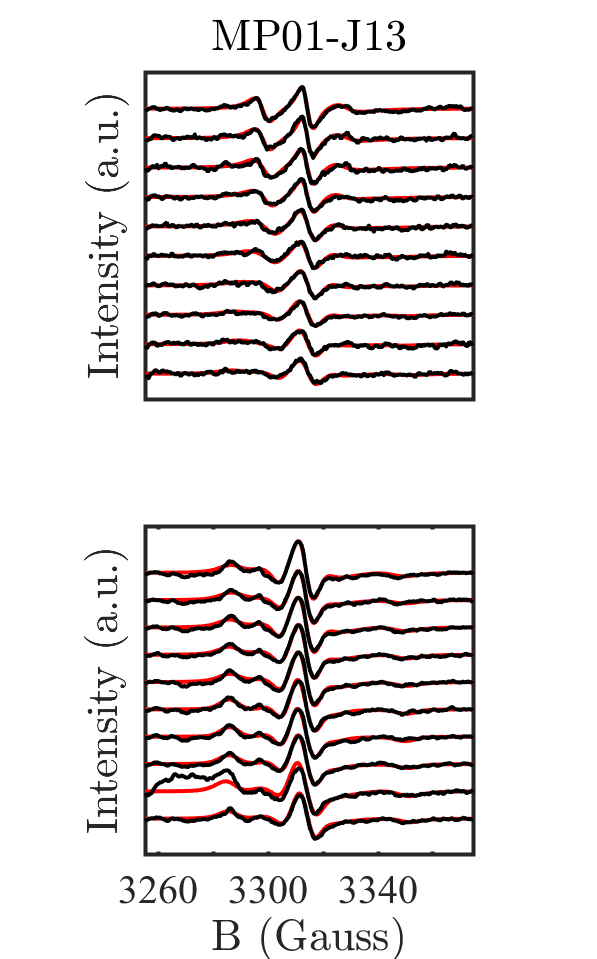


**Figure S7.** EPR spectra and best fits for unreacted (top) and reacted (bottom) MP01-J13. One of the spectra of the reacted molecule has an aberration at low magnetic fields, which resulted from a sample alignment issue. This did not appear to affect fitting.


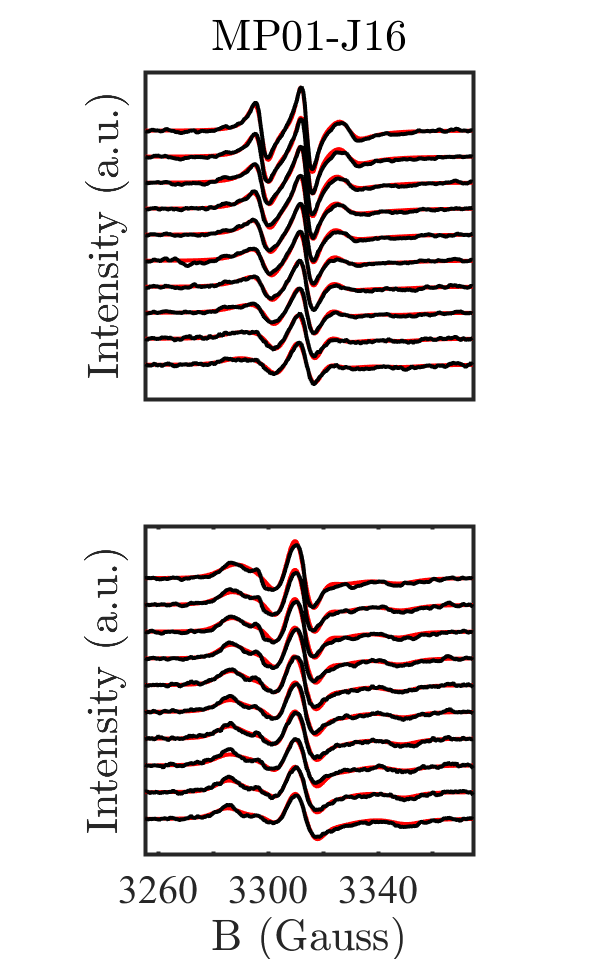


**Figure S8.** EPR spectra and best fits for unreacted (top) and reacted (bottom) MP01-J13.


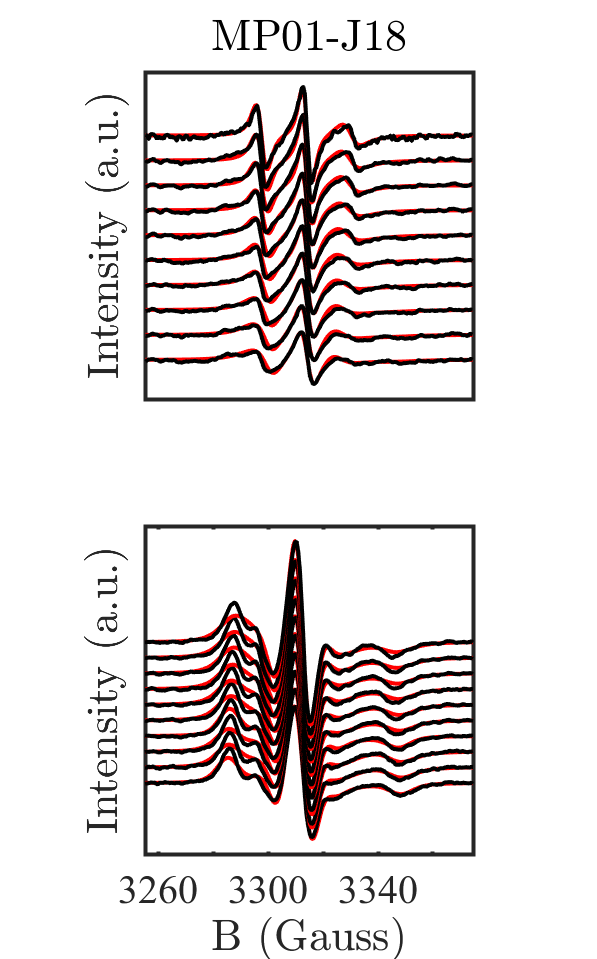


**Figure S9.** EPR spectra and best fits for unreacted (top) and reacted (bottom) MP01-J18.


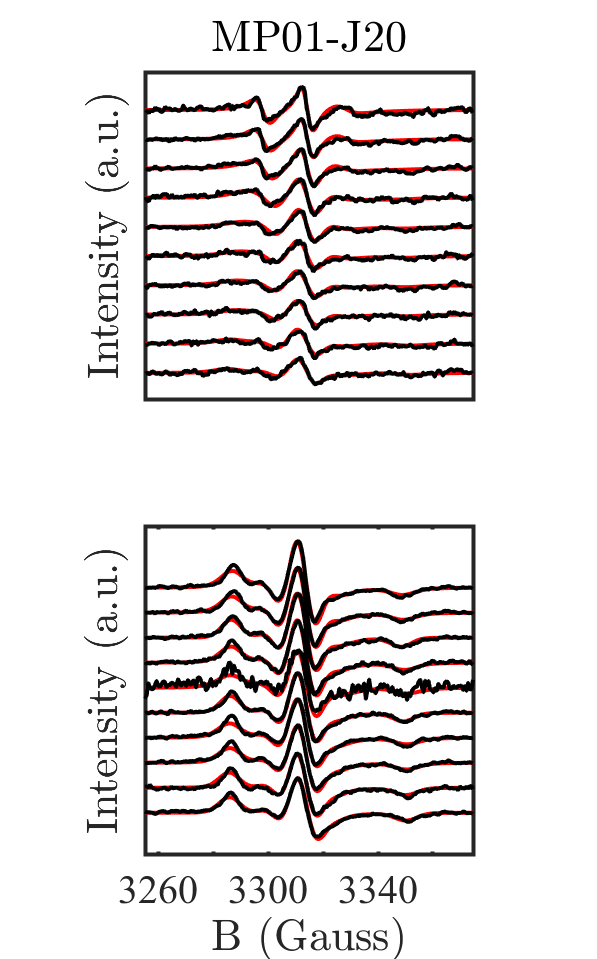


**Figure S10.** EPR spectra and best fits for unreacted (top) and reacted (bottom) MP01-J20.The large noise present in one of the reacted spectra resulted from an alignment issue, and did not appear to affect fitting.


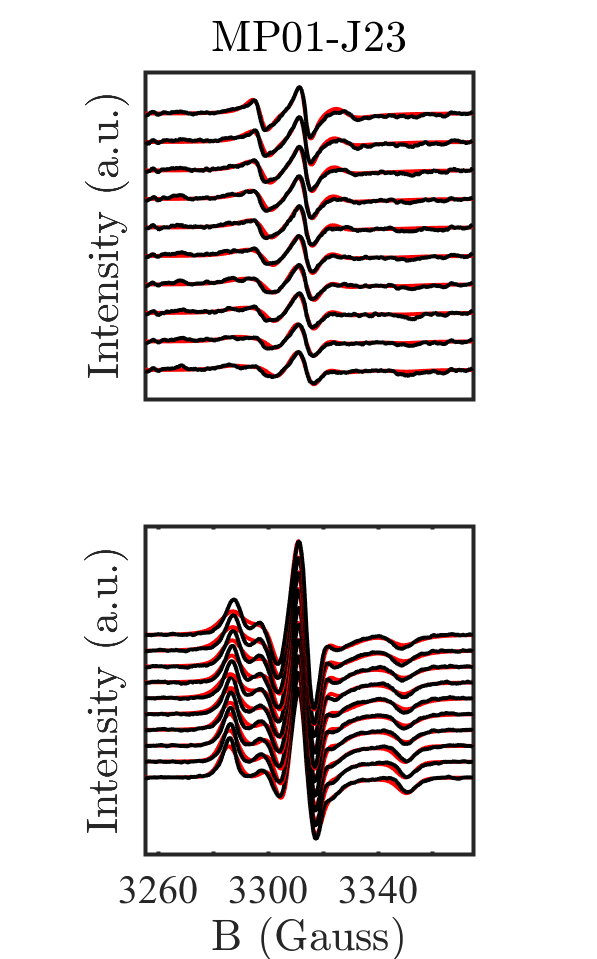


**Figure S11.** EPR spectra and best fits for unreacted (top) and reacted (bottom) MP01-J23.


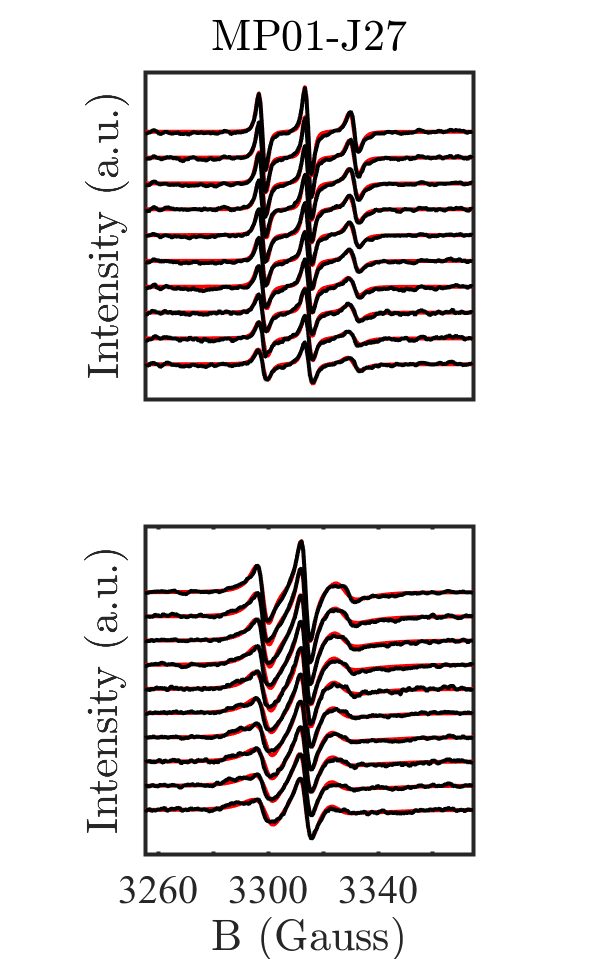


**Figure S12.** EPR spectra and best fits for unreacted (top) and reacted (bottom) MP01-J27.


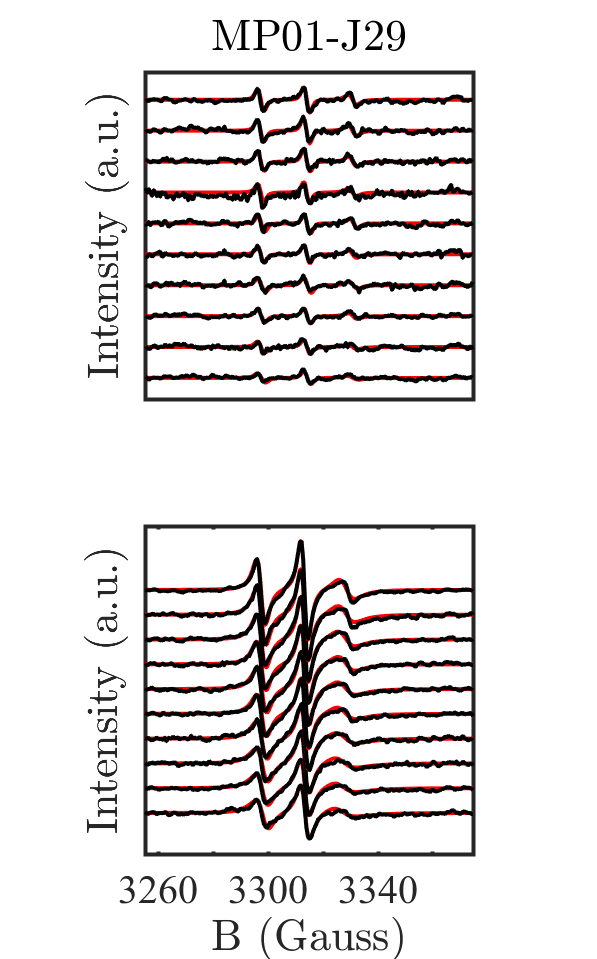


**Figure S13.** EPR spectra and best fits for unreacted (top) and reacted (bottom) MP01-J29. The unreacted peptides have very low spectral intensity because they were not reacted with K_3_Fe(CN)­_6_, since LCMS demonstrated that this damaged unreacted MP01-J29. Consequently, the bulk of the MP01-J29 existed in the spectrally inert hydroxlyaminated form, giving very low-intensity spectra. Consequently, noise heavily affected these fits, resulting in a wide range of fitting error.

# 4. Mass Spectra of Unreacted Peptides


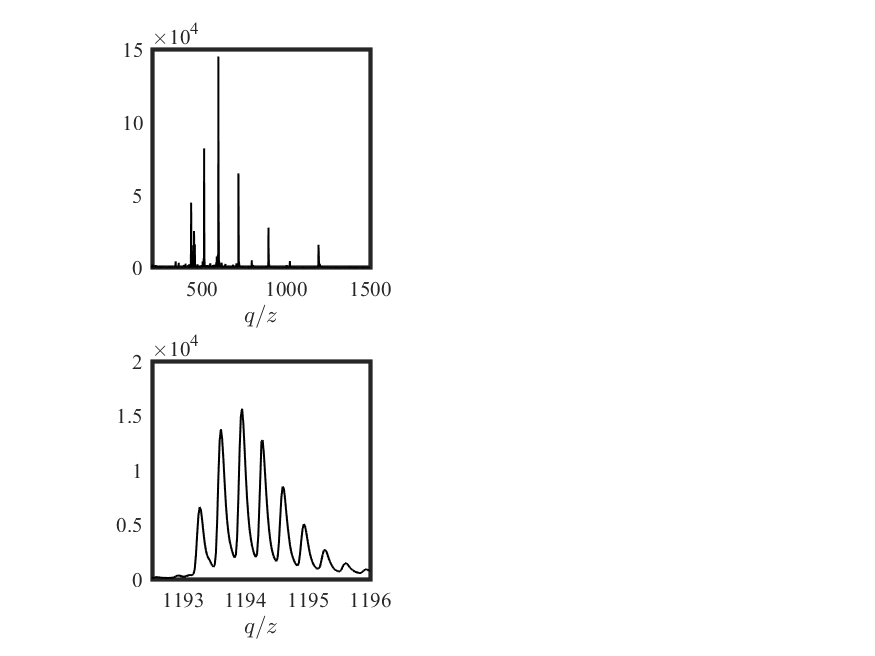


**Figure S14.** The LC-MS spectrum of major peaks of the MP01-J3 chromatography curve. A feature of the [M+3H]^3+^ charge state used for mass calculation is shown beneath. For predicted and calculated masses, refer to Table S1.


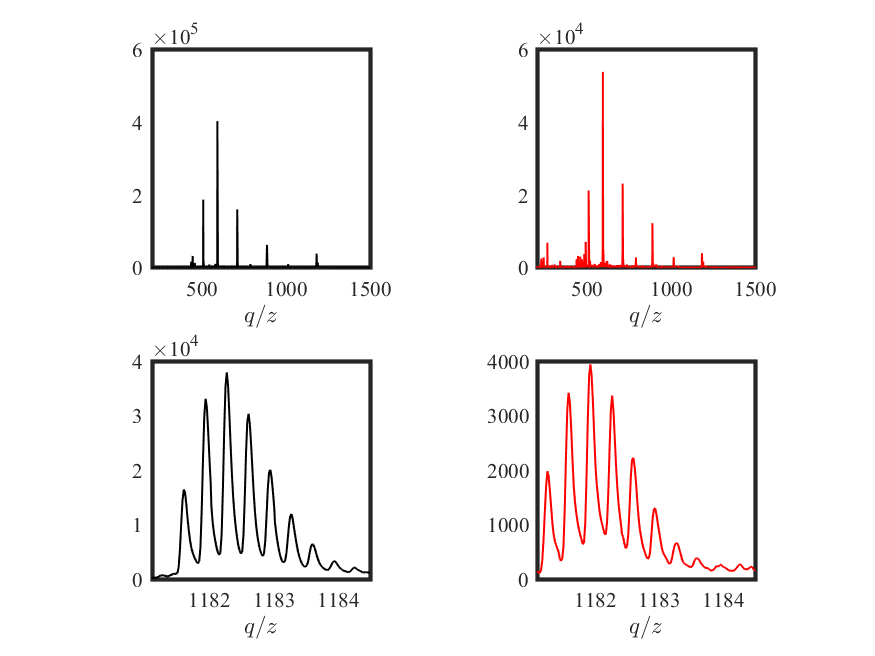


**Figure S15.** The LC-MS spectrum of major peaks of the MP01-J5 chromatography curve. A feature of the [M+3H]^3+^ charge state used for mass calculation is shown beneath. The (major) hydroxylamine product is shown in black, and the (minor) nitroxide product is shown in red. For predicted and calculated masses, refer to Table S1.


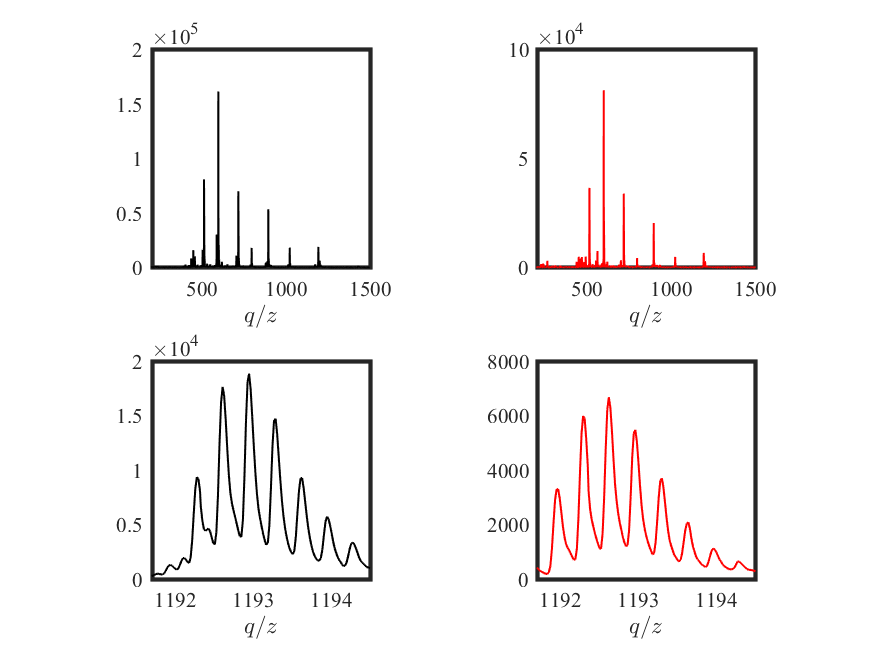


**Figure S16.** The LC-MS spectrum of major peaks of the MP01-J7 chromatography curve. A feature of the [M+3H]^3+^ charge state used for mass calculation is shown beneath. The (major) hydroxylamine product is shown in black, and the (minor) nitroxide product is shown in red. For predicted and calculated masses, refer to Table S1.


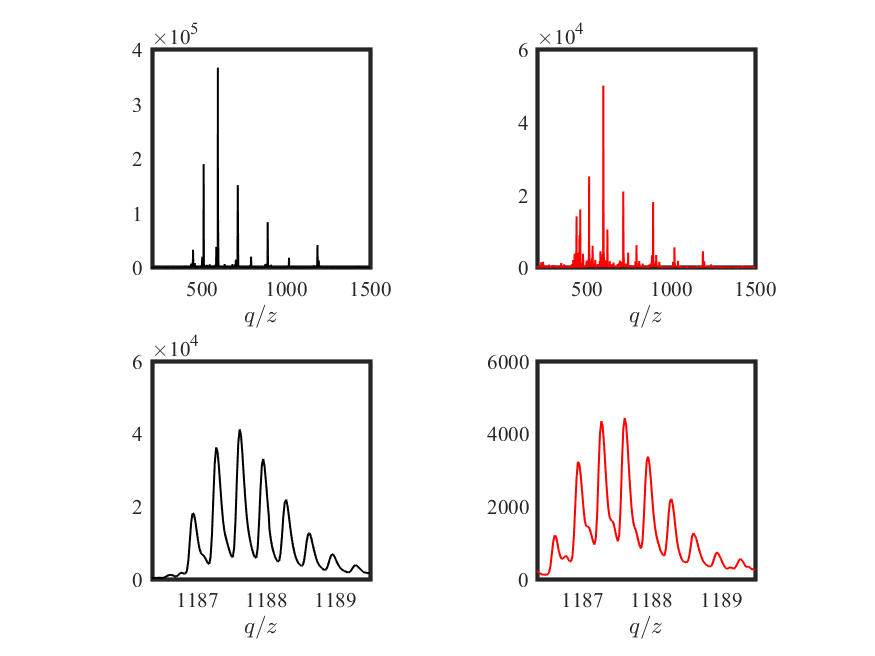


**Figure S17.** The LC-MS spectrum of major peaks of the MP01-J13 chromatography curve. A feature of the [M+3H]^3+^ charge state used for mass calculation is shown beneath. The (major) hydroxylamine product is shown in black, and the (minor) nitroxide product is shown in red. For predicted and calculated masses, refer to Table S1.


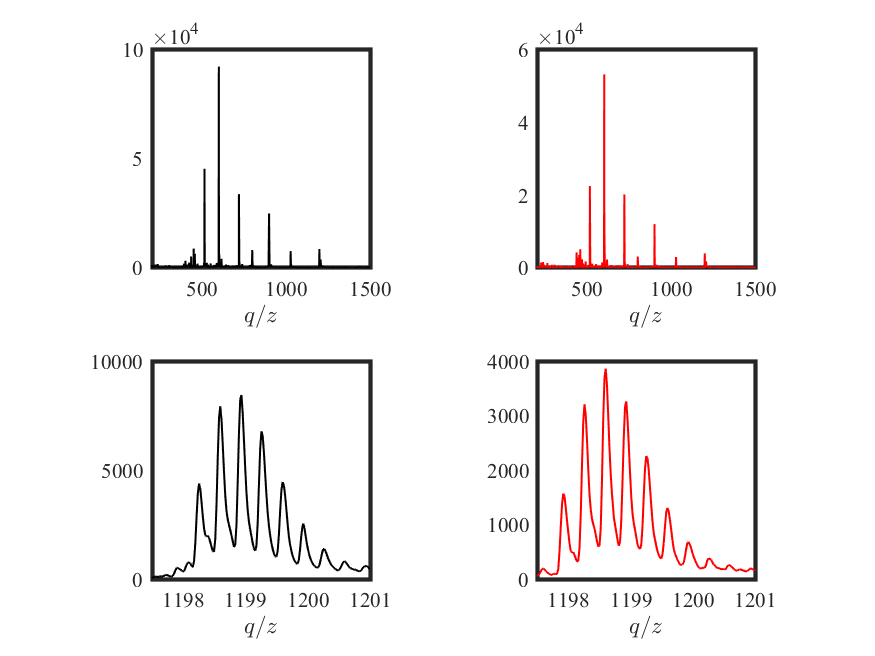


**Figure S18.** The LC-MS spectrum of major peaks of the MP01-J16 chromatography curve. A feature of the [M+3H]^3+^ charge state used for mass calculation is shown beneath. The (major) hydroxylamine product is shown in black, and the (minor) nitroxide product is shown in red. For predicted and calculated masses, refer to Table S1. Smaller peaks in these spectra originate from a minor, dimerized peptide.


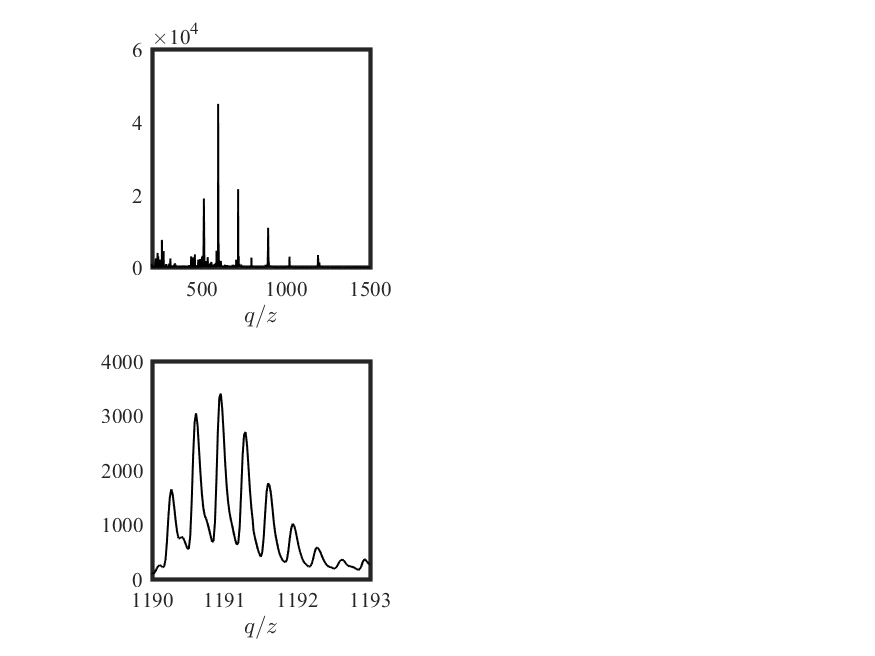


**Figure S19.** The LC-MS spectrum of major peaks of the MP01-J18 chromatography curve. A feature of the [M+3H]^3+^ charge state used for mass calculation is shown beneath. For predicted and calculated masses, refer to Table S1.


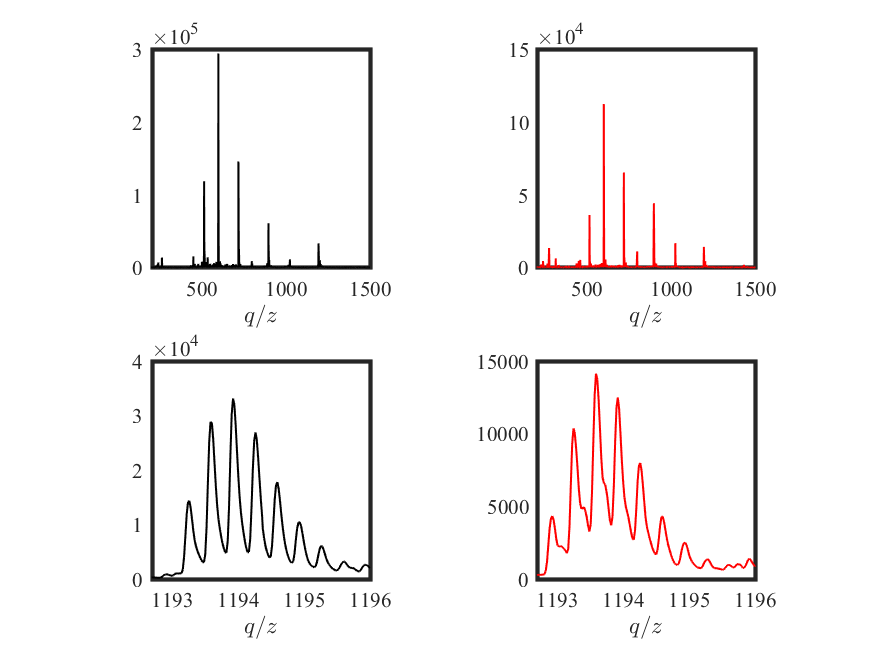


**Figure S20.** The LC-MS spectrum of major peaks of the MP01-J20 chromatography curve. A feature of the [M+3H]^3+^ charge state used for mass calculation is shown beneath. The (major) hydroxylamine product is shown in black, and the (minor) nitroxide product is shown in red. For predicted and calculated masses, refer to Table S1. Smaller peaks in the hydroxylamine signal originate from a minor, dimerized peptide.


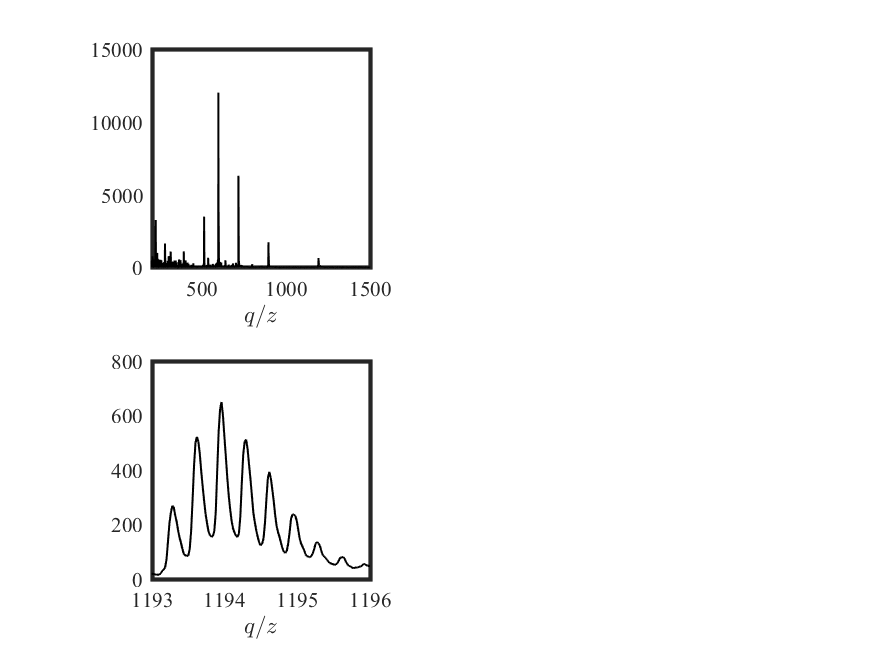


**Figure S21.** The LC-MS spectrum of major peaks of the MP01-J23 chromatography curve. A feature of the [M+3H]^3+^ charge state used for mass calculation is shown beneath. For predicted and calculated masses, refer to Table S1.


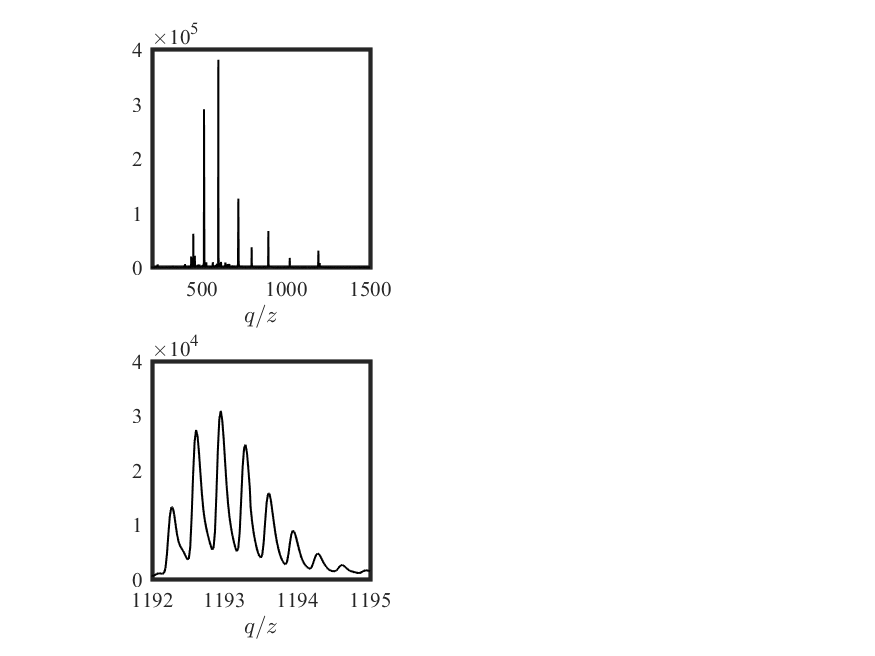
**Figure S22.** The LC-MS spectrum of major peaks of the MP01-J27 chromatography curve. A feature of the [M+3H]^3+^ charge state used for mass calculation is shown beneath. For predicted and calculated masses, refer to Table S1. Smaller peaks in the hydroxylamine spectrum originate from a minor, dimerized peptide.


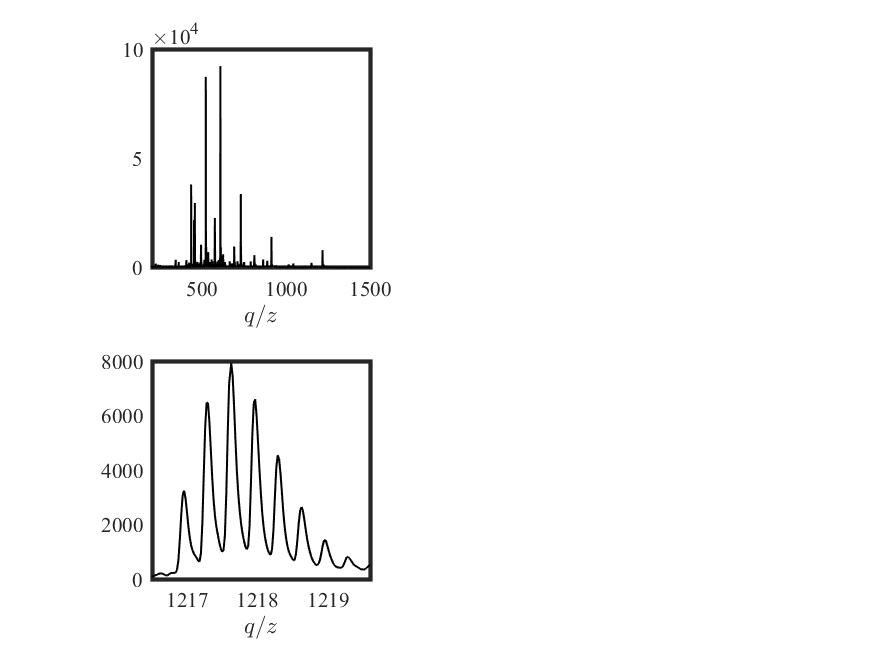
**Figure S23.** The LC-MS spectrum of major peaks of the MP01-J29 chromatography curve. A feature of the [M+3H]^3+^ charge state used for mass calculation is shown beneath. For predicted and calculated masses, refer to Table S1. The dominant set of impurity peaks in the hydroxylamine spectrum originate from a TOAC deletion product, which is EPR-invisible.

# 5. Mass Spectra of Reacted Peptides


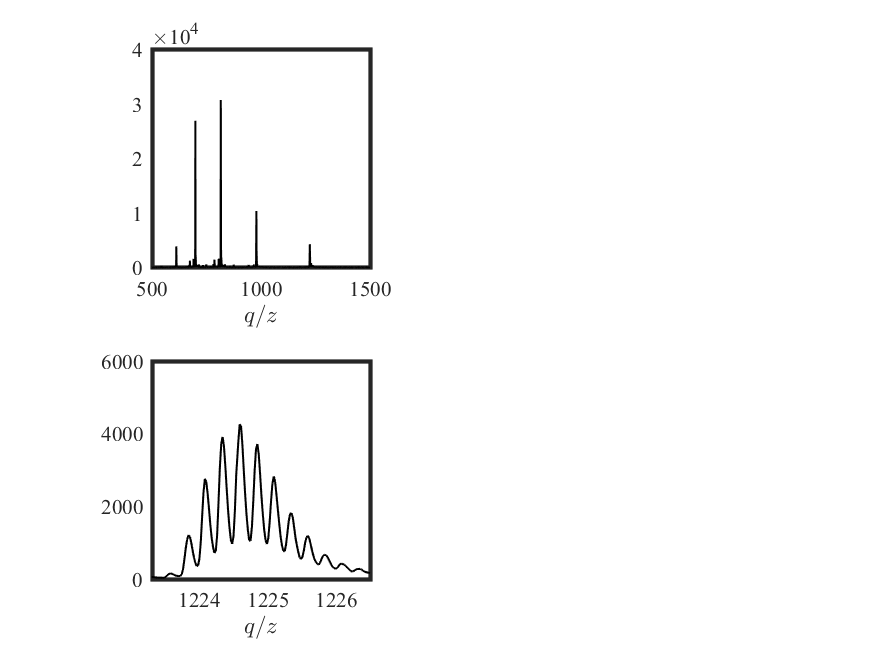


**Figure S24.** The LC-MS spectrum of major peaks of the labeled MP01-J3 chromatography curve. A feature of the [M+4H]^4+^ charge state used for mass calculation is shown beneath. For predicted and calculated masses, refer to Table S1.


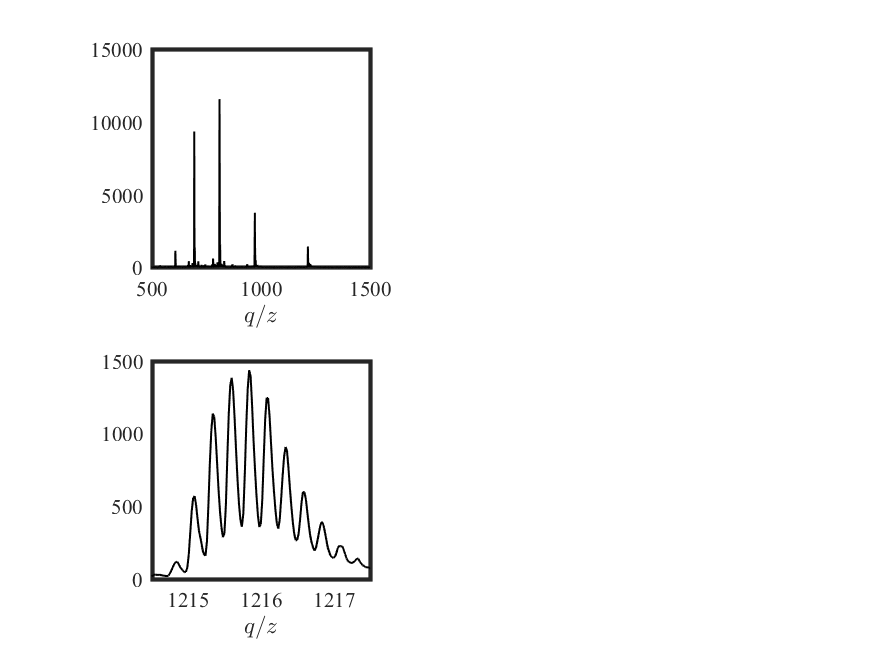


**Figure S25.** The LC-MS spectrum of major peaks of the labeled MP01-J5 chromatography curve. A feature of the [M+4H]^4+^ charge state used for mass calculation is shown beneath. Here the nitroxide peaks overlap the hydroxylamine peak, explaining the first peak in the [M+4H]^4+^ feature. For predicted and calculated masses, refer to Table S1.


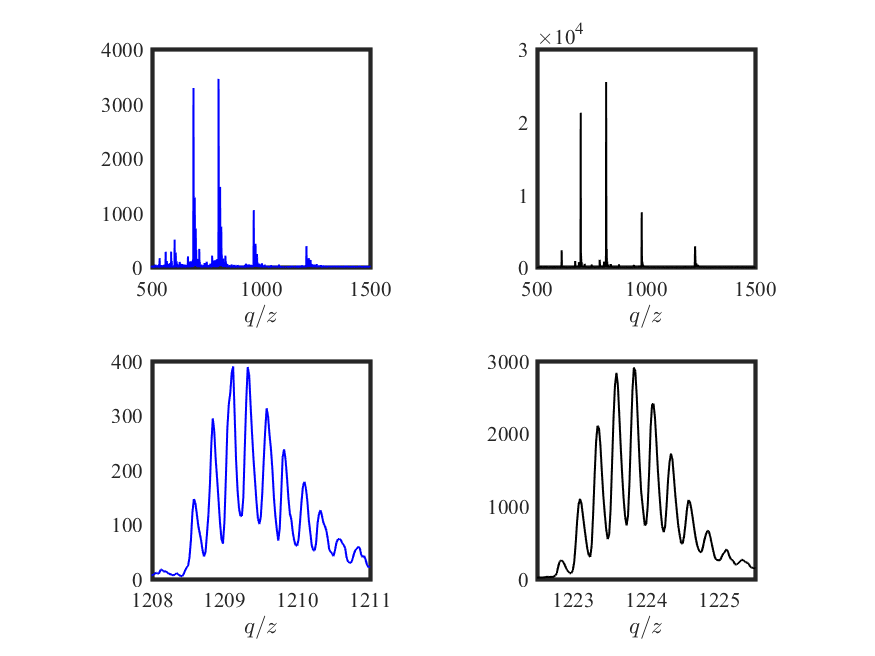


**Figure S26.** The LC-MS spectrum of major peaks of the labeled MP01-J7 chromatography curve. A feature of the [M+4H]^4+^ charge state used for mass calculation is shown beneath. The (major) hydroxylamine product is shown in black and the (minor) nitroxylated glycine deletion product is shown in blue. For predicted and calculated masses, refer to Table S1.


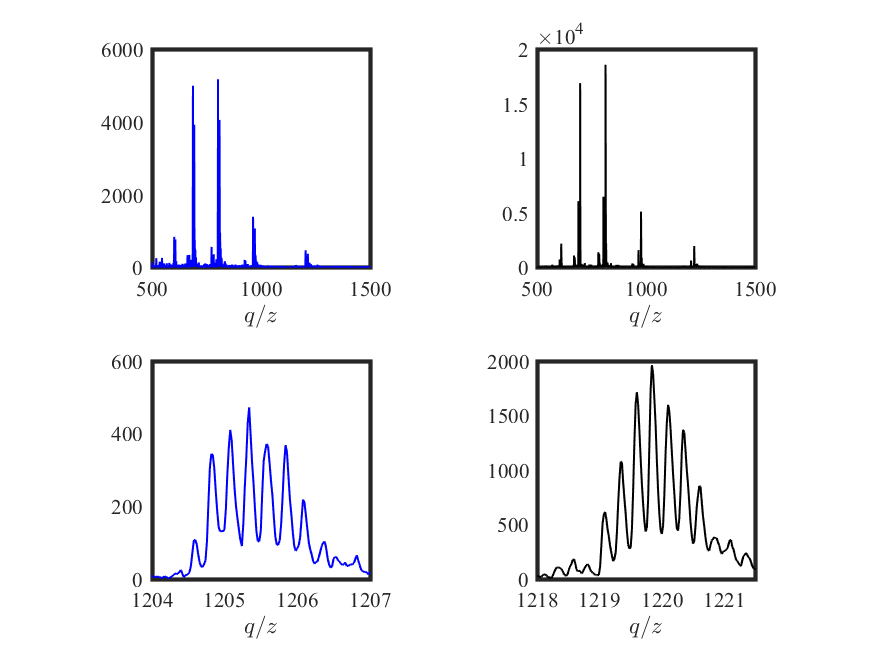


**Figure S27.** The LC-MS spectrum of major peaks of the labeled MP01-J13 chromatography curve. A feature of the [M+4H]^4+^ charge state used for mass calculation is shown beneath. The (major) hydroxylamine product is shown in black and the (minor) nitroxylated glycine deletion product is shown in blue. For predicted and calculated masses, refer to Table S1.


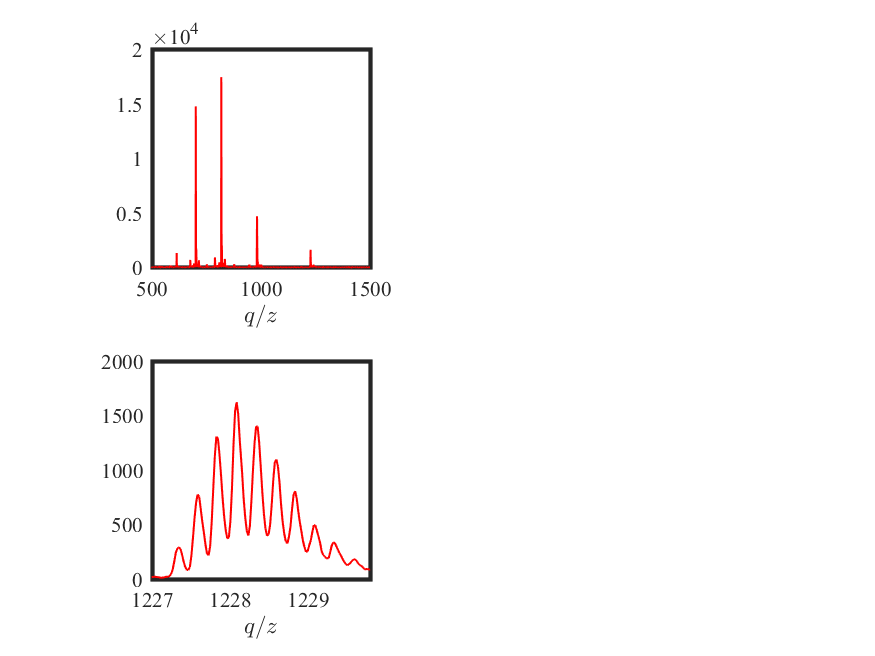


**Figure S28.** The LC-MS spectrum of major peaks of the labeled MP01-J16 chromatography curve. A feature of the [M+4H]^4+^ charge state used for mass calculation is shown beneath. In this case, the dominant product appears to be the nitroxyl radical, so the corresponding signal is plotted in red. For predicted and calculated masses, refer to Table S1.


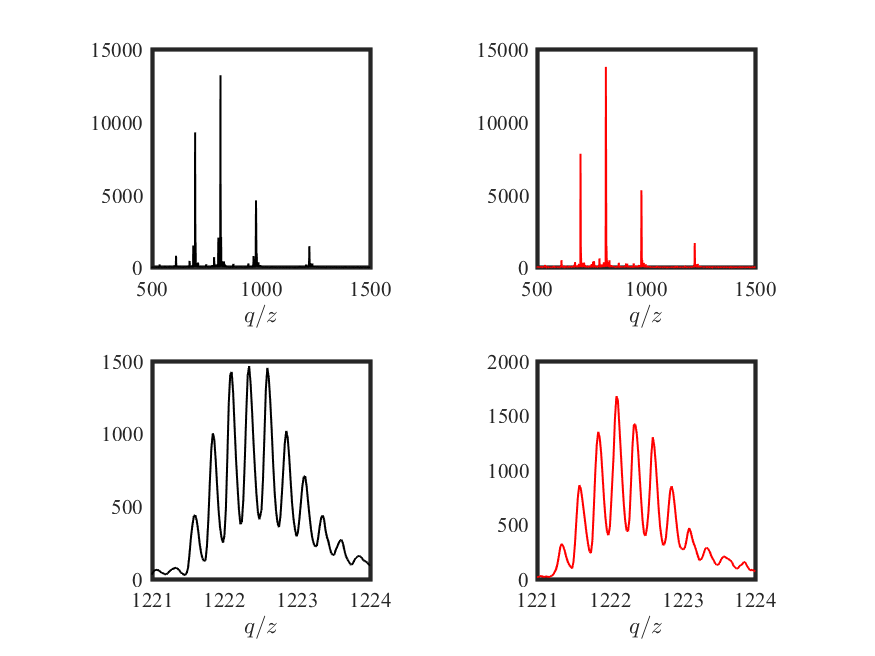


**Figure S29.** The LC-MS spectrum of major peaks of the labeled MP01-J18 chromatography curve. A feature of the [M+4H]^4+^ charge state used for mass calculation is shown beneath. The (major) hydroxylamine product is shown in black, and the (minor) nitroxide product is shown in red. For predicted and calculated masses, refer to Table S1.


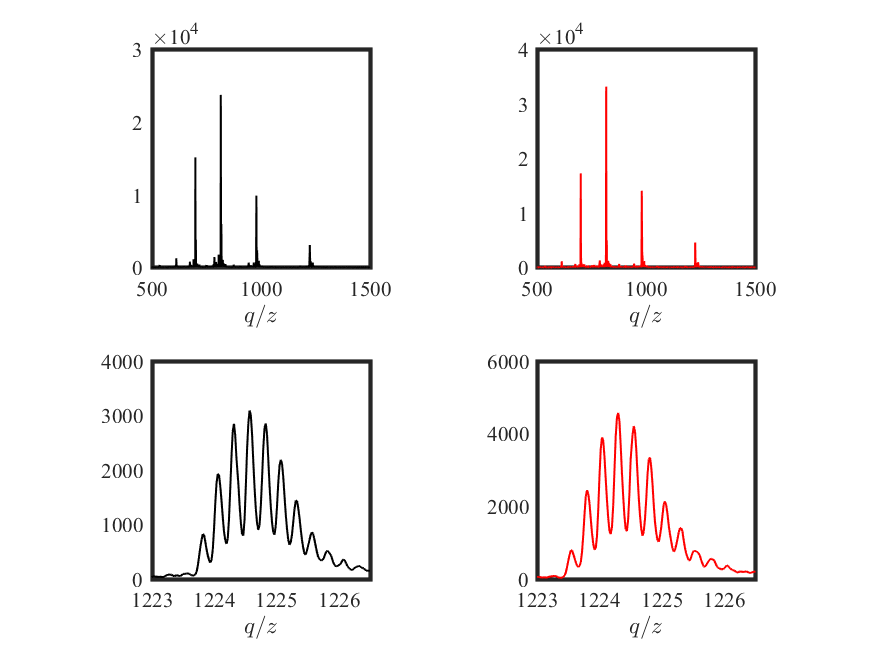


**Figure S30.** The LC-MS spectrum of major peaks of the labeled MP01-J20 chromatography curve. A feature of the [M+4H]^4+^ charge state used for mass calculation is shown beneath. The (major) hydroxylamine product is shown in black, and the (minor) nitroxide product is shown in red. For predicted and calculated masses, refer to Table S1.


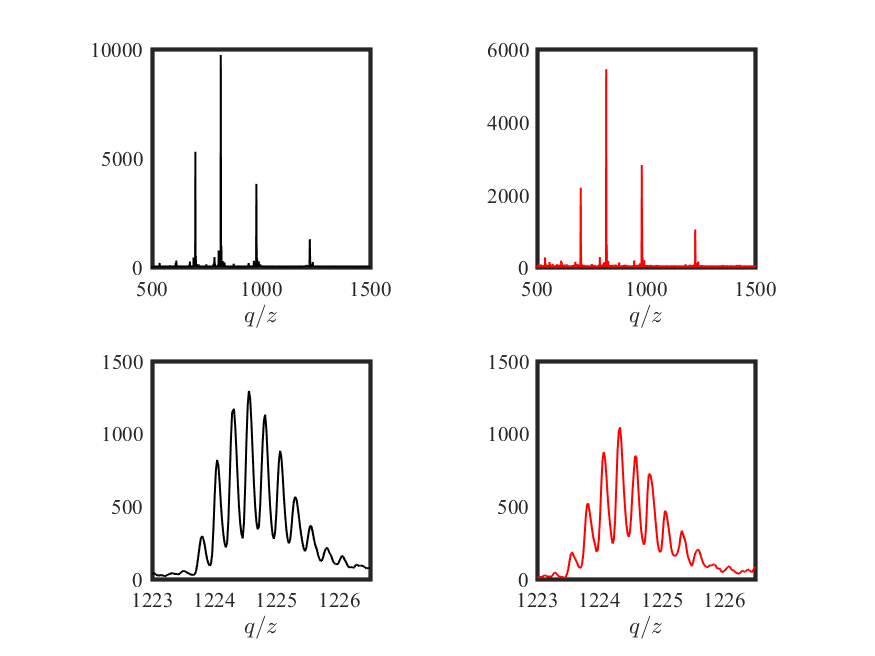


**Figure S31.** The LC-MS spectrum of major peaks of the labeled MP01-J23 chromatography curve. A feature of the [M+4H]^4+^ charge state used for mass calculation is shown beneath. The (major) hydroxylamine product is shown in black, and the (minor) nitroxide product is shown in red. For predicted and calculated masses, refer to Table S1.


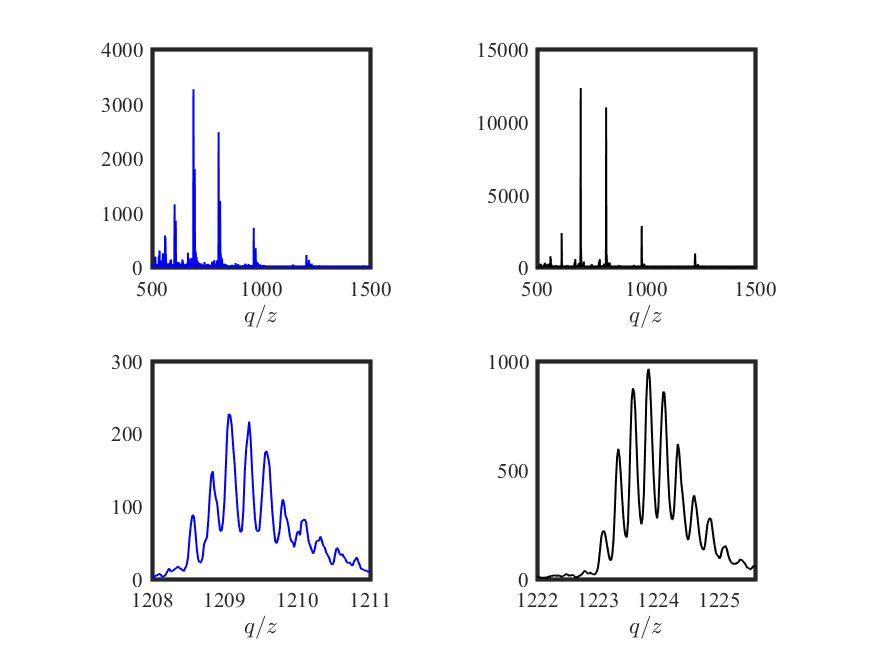


**Figure S32.** The LC-MS spectrum of major peaks of the labeled MP01-J27 chromatography curve. A feature of the [M+4H]^4+^ charge state used for mass calculation is shown beneath. The (major) hydroxylamine product is shown in black and the (minor) nitroxylated glycine deletion product is shown in blue. For predicted and calculated masses, refer to Table S1.


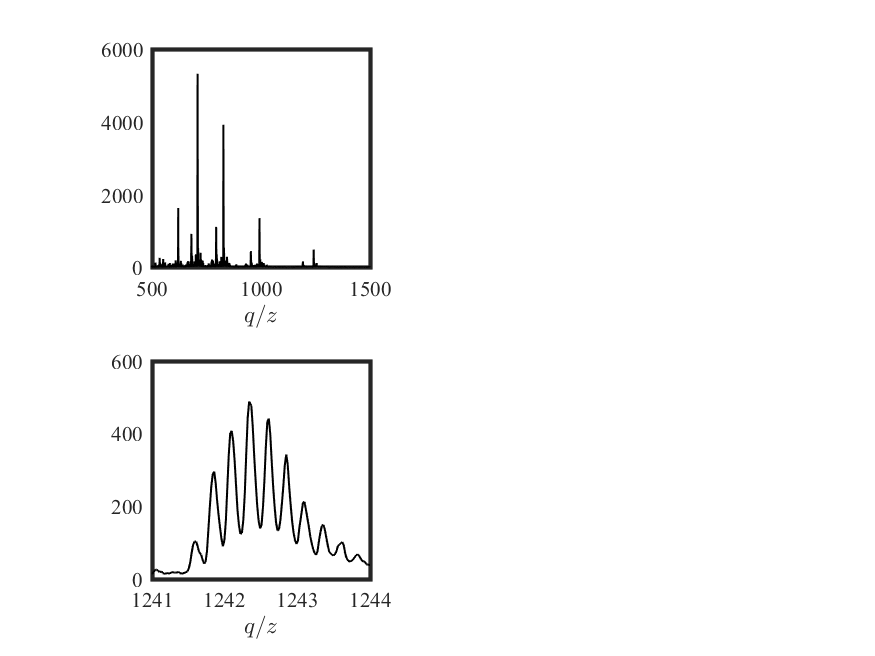


**Figure S32.** The LC-MS spectrum of major peaks of the labeled MP01-J27 chromatography curve. A feature of the [M+4H]^4+^ charge state used for mass calculation is shown beneath. The smaller set of peaks visible in the signal originate from the TOAC deletion product, to which EPR measurement is insensitive. For predicted and calculated masses, refer to Table S1.

# 6. References

(1) Evans, E. D.; Pentelute, B. L. Discovery of a 29-Amino-Acid Reactive Abiotic Peptide for Selective Cysteine Arylation. *ACS Chem. Biol.* **2018**, *13*, 527–532 . https://doi.org/10.1021/acschembio.7b00520.

(2) Slezak, D. F.; Suarez, C.; Cecchi, G. A.; Marshall, G.; Stolovitzky, G. When the Optimal Is Not the Best : Parameter Estimation in Complex Biological Models. *PLoS One* **2010**, *5* (10), e13283 . https://doi.org/10.1371/journal.pone.0013283.

(3) Budil, D. E.; Lee, S.; Saxena, S.; Freed, J. H. Nonlinear-Least-Squares Analysis of Slow-Motion EPR Spectra in One and Two Dimensions Using a Modified Levenberg – Marquardt Algorithm. *J. Magn. Reson.* **1996**, *120*, 155–189.
